# Charge and heat transport in soft nanosystems in the presence of time-dependent perturbations

**DOI:** 10.3762/bjnano.7.39

**Published:** 2016-03-18

**Authors:** Alberto Nocera, Carmine Antonio Perroni, Vincenzo Marigliano Ramaglia, Vittorio Cataudella

**Affiliations:** 1 Department of Physics, Northeastern University, Boston, MA 02115, USA; 2 CNR-SPIN and Department of Physics “Ettore Pancini”, Universita’ degli Studi di Napoli Federico II, Complesso Universitario Monte Sant’Angelo, Via Cintia, I-80126 Napoli, Italy

**Keywords:** electronic charge quantum pumping, electronic transport theory, nanoelectromechanical systems, thermoelectric properties, time-dependent perturbations

## Abstract

**Background:** Soft nanosystems are electronic nanodevices, such as suspended carbon nanotubes or molecular junctions, whose transport properties are modulated by soft internal degrees of freedom, for example slow vibrational modes. Effects of the electron–vibration coupling on the charge and heat transport of soft nanoscopic systems are theoretically investigated in the presence of time-dependent perturbations, such as a forcing antenna or pumping terms between the leads and the nanosystem. A well-established approach valid for non-equilibrium adiabatic regimes is generalized to the case where external time-dependent perturbations are present. Then, a number of relevant applications of the method are reviewed for systems composed by a quantum dot (or molecule) described by a single electronic level coupled to a vibrational mode.

**Results:** Before introducing time-dependent perturbations, the range of validity of the adiabatic approach is discussed showing that a very good agreement with the results of an exact quantum calculation is obtained in the limit of low level occupation. Then, we show that the interplay between the low frequency vibrational modes and the electronic degrees of freedom affects the thermoelectric properties within the linear response regime finding out that the phonon thermal conductance provides an important contribution to the figure of merit at room temperature. Our work has been stimulated by recent experimental results on carbon nanotube electromechanical devices working in the semiclassical regime (resonator frequencies in the megahertz range compared to an electronic hopping frequency of the order of tens of gigahertz) with extremely high quality factors. The nonlinear vibrational regime induced by the external antenna in such systems has been discussed within the non-perturbative adiabatic approach reproducing quantitatively the characteristic asymmetric shape of the current–frequency curves. Within the same set-up, we have proved that the antenna is able to pump sufficient charge close to the mechanical resonance making single-parameter adiabatic charge pumping feasible in carbon nanotube resonators. The pumping mechanism that we observe is different from that acting in the two parameter pumping and, instead, it is based on an important dynamic adjustment of the mechanical motion of the nanotube to the external drive in the weakly nonlinear regime. Finally, stochastic forces induced by quantum and thermal fluctuations due to the electron charging of the quantum dot are shown to affect in a significant way a Thouless charge pump realized with an elastically deformable quantum dot. In this case, the pumping mechanism is also shown to be magnified when the frequency of the external drive is resonant with the proper frequency of the deformable quantum dot. In this regime, the pumping current is not strongly reduced by the temperature, giving a measurable effect.

**Conclusion:** Aim of this review has been to discuss common features of different soft nanosystems under external drive. The most interesting effects induced by time-dependent perturbations are obtained when the external forcing is nearly resonant with the slow vibrational modes. Indeed, not only the external forcing can enhance the electronic response, but it also induces nonlinear regimes where the interplay between electronic and vibrational degrees of freedom plays a major role.

## Introduction

In some nanoelectronic devices, internal soft degrees of freedom, such as slow vibrational modes, cannot be neglected since they actively modulate the transport properties. Indeed, the electron–vibration coupling significantly affects the charge and heat transport of nanoscopic devices such as molecules connected to external leads [1–4], and nanoelectromechanical systems [5–8].

Due to the small dimensions of the molecular bridge, the hopping of an electron from the lead onto the molecule can significantly alter its nuclear configuration. As a main consequence, intriguing nonlinear phenomena, such as hysteresis, switching, and negative differential conductance have been observed in molecular junctions. In conducting molecules, either the center of mass oscillations [9], or thermally induced acoustic phonons [10] can be the source of coupling between electronic and vibrational degrees of freedom.

Nanoelectromechanical systems (NEMS) are devices similar to molecular junctions. Typically, they consist of a nanobeam resonator that is coupled to an electronic quantum dot junction. Famous examples of NEMS are suspended carbon nanotube (CNT) resonators, which are anchored to two metallic leads under bias voltage. In this case, the quantum dot is embedded in the CNT itself. In [11–12] the motion of the CNT is actuated by a nearby antenna, which means that, when the external antenna frequency matches the natural frequency of the CNT beam, one can measure the CNT oscillation frequency from the electronic current response of the device. This is possible due to the extremely high quality factors (*Q >* 10^5^) observed when the resonator frequencies fall in the megahertz range compared with an electronic hopping frequency from the leads of the order of tens of gigahertz. Recently, it has been found that phenomena such as switching, hysteresis, as well as multistability can be observed in NEMS [13]. NEMS have been proposed as high sensitive position and mass sensors [14–20].

Recently, research at the nanoscale has focused not only on charge but also on heat transport [21–24]. In particular, thermopower and thermal conductances have been measured and theoretically calculated in molecular junctions [25–29]. The role of vibrational degrees of freedom and their coupling with electrons have a fundamental importance on heat transport and dissipation. Moreover, the effect of time perturbations not only on the charge dynamics but also on the energy transport is becoming a new field of study where both electronic and vibrational degrees of freedom are involved [30–35].

It has been shown that periodic time-dependent perturbations can lead to pumping effects depending on the frequency of the external drive. In this context, different setups have been studied. Recent experiments have shown the possibility of realizing single-parameter charge pumping [36–39] on devices similar to those described above. However, the characteristic frequencies of the external drive are much larger than the electronic tunneling rate. In fact, in these conditions, an effective phase-shift can be produced due to electron–electron interactions, which is intrinsically generated by a non-adiabatic blockade of tunneling [40]. However, it has been pointed out that higher frequencies are necessary to observe pumping currents of the same order of magnitude of those observed when a two-parameter pumping mechanism is present [41–45]. Another particularly interesting experiment has been carried out in [46], where charge conductance has been obtained at zero bias voltage, by applying a small power to the antenna at a frequency close to that of the internal slow resonator. Therefore, it is of great importance to address theoretically the single-parameter charge pumping in the regime where the driving frequencies are smaller than the electronic tunneling rates of the device and close to the frequency of the internal vibrational mode. Indeed, in the absence of an internal degree of freedom, it has been theoretically demonstrated that single-parameter charge pumping through a quantum dot in the this regime is poor [47] even if the electronic correlations are important [48–49].

The relative magnitude of the characteristic vibrational frequency of the molecule or nanobeam and the hopping rate of electrons from the leads represents an important quantity in understanding the physics of molecular junctions and NEMS. In this review, we analyze the adiabatic regime, realized when the internal vibrational modes have frequencies smaller than the hopping rate. Within this regime, one can observe phenomena such as switching, multistability and hysteresis in molecular junctions or NEMS, and study the physics of NEMS subjected to periodic perturbations such those described above. When the transient dynamics is considered, and the uniqueness and the approach to the steady state is concerned, analytical and numerical approaches have given controversial results. This is because of the length of the time scales required to reach a steady state (if a steady state is reached at all) [50–54]. A strong debate has developed in the literature regarding the existence of multistability in these systems [52–54] by studying models similar to those investigated in this review. Although we do not address this issue here, in our approach multistability is not found.

The electron–vibration coupling within the Anderson–Holstein model has received much theoretical attention both in the non-adiabatic [55–61] and in the adiabatic regime. In this latter case, which is the focus of our review, it has been studied in a fully out-of-equilibrium response regime with different theoretical tools, ranging from rate equations [62–66] to non-equilibrium Green’s function formalisms [48,55,67–69].

The adiabatic approach, which has been applied by some of the present authors in other contexts [70], is based on the time-scale separation between the slow dot vibrational degrees of freedom and the fast electronic time scales involved in the thermal or charge transport. In [68,71–72], the case of a single vibrational mode within the Anderson–Holstein model has been studied with the Feynman–Vernon action functional formalism in the adiabatic regime. We stress that, within the adiabatic approach, the coupling between electrons and vibrational degrees of freedom can be arbitrarily large. On the other hand, the approach followed in [72] is valid for electronic and vibrational time scales of the same order of magnitude, but it is fundamentally correct only in the regime of weak electron–vibration and vibration–vibration coupling (negligible anharmonicity). The focus of this review will be on non-perturbative electron–vibration and vibration–vibration coupling regimes.

We point out that the adiabatic approach discussed in this review is somewhat different from methods based on the Ehrenfest dynamics [73–74]. Indeed, from the point of view of the electronic system, the adiabatic approach and the Ehrenfest dynamics are at the same level of approximation: The electron dynamics is treated considering the vibrational degrees of freedom classical and infinitely slow. However, from the point of view of the vibrational dynamics, the Ehrenfest approach is poorer than adiabatic approach. Actually, the Ehrenfest approach is similar to a mean-field approximation where not the correct force but the spatial derivative of the mean value of the electronic Hamiltonian is derived. Moreover, in the Ehrenfest approach, no dissipative and fluctuating terms are evaluated for the vibrational dynamics. These terms in the adiabatic approach are extremely important: They satisfy the fluctuation–dissipation relation at the equilibrium, and they include the semiclassical corrections fundamental to treat the out-of-equilibrium regime. Of course, the Ehrenfest dynamics can be combined in a simpler way with ab initio calculations [75] of the electronic and/or vibrational systems. But this is not the focus of our review, which is based on model Hamiltonians for both electronic and vibrational degrees of freedom.

In the absence of an external periodic perturbation and in the limit of small vibrational frequency with respect to electronic hopping, a generalized Langevin equation for the displacement coordinate of the vibrational mode can be derived. In [76], we have shown that the same Langevin equation can be obtained if one performs a semiclassical approximation on the model Hamiltonian and carries out an adiabatic approximation directly in the electronic Green’s function on the Keldysh contour. The semiclassical approach naturally includes the effect of a noise term that stems form the quantum charge fluctuations induced by the fast time scales of the electronic system. The friction and the noise strengths depend by construction on the displacement of the oscillator from the equilibrium position. Our semiclassical approximation, even if less general than the influence functional approach, allows us to disentangle exactly the quantum effects of the dynamics of the oscillator in the Langevin equation and is valid for an arbitrary strength of electron–vibration coupling [76–77].

In this review, the adiabatic approach for the local vibrational degrees of freedom in soft nanoscopic systems has been generalized to the case where external time-dependent periodic perturbations, such as the effects of a forcing antenna and pumping terms between the leads and the nanoscopic system, are present. In the absence of temporal perturbations, we have also included the presence of phononic baths, which is important when thermal transport through the nanosystem is addressed. Even if the approach can be applied to multilevel electronic systems with an arbitrary number of vibrational degrees of freedom, in this review, we have mostly discussed the results corresponding to the prototype system composed of a quantum dot or a molecule described by a single electronic level coupled to a single vibrational mode.

Before introducing temporal perturbations, we have thoroughly studied the range of validity of the approach, focusing first on the case of zero bias voltage at any temperature, then on the case of zero temperature at finite bias voltages. The thermoelectric properties have then been analyzed within the linear response regime focusing on the phonon thermal contribution 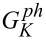 to the figure of merit *ZT* at room temperature. Parameters appropriate for junctions based on C_60_ molecules connected between different metallic leads have been considered for the thermoelectric transport. We have finally generalized the treatment of the heat transport to the case where also electron–electron interaction on the dot is present (within the Coulomb blockade regime) and generalized the adiabatic approach to this case.

Then, we have analyzed the properties of the single dot in the presence of time-dependent periodic perturbations, and in particular of an external forcing antenna. We have included the effect of the forcing antenna in our adiabatic scheme showing that the resulting Langevin equation for the vibrational mode is modified by a periodic forcing term. Moreover, the generalized force term, the friction and the noise strengths become functions that depend on the oscillator displacement and acquire an explicit periodic dependence on time.

We have treated distinct systems in our unified approach. In particular we have studied the electronic transport properties at finite bias of a NEMS device consisting of a vibrating suspended CNT actuated by an external antenna. In this setup, we show that a single-parameter charge pumping is possible. In particular, when the frequency of the antenna is close to that of the oscillating nanotube, the amplification of the mechanical response generates an intrinsic imbalance in the response of the current within a single pumping cycle, giving a net non-zero contribution. Interestingly, we have found theoretically that, in the nonlinear regime, the pumping current has a non-zero response also to harmonics higher than one and their behaviour has been compared with the features of the first harmonic.

Finally, we have investigated the behaviour of a two-parameter quantum dot device (Thouless pump) in the presence of an internal vibrational degree of freedom. The characteristics of the pumping current have been studied as a function of the electron–vibration coupling, temperature, and driving frequency. We have assumed that both the frequency of the driving forces and the intrinsic oscillation frequency of the quantum dot vibration are adiabatic. We have found theoretically that the pumping current can be amplified by the internal vibration of the quantum dot. As found above, this is possible only when the frequency of the driving forces are close to resonance with the vibration of the dot. We have shown that the amplification is robust against temperature, leading to the prediction that, different from expectation, slowly oscillating quantum dots could have pumping effects measurable up to high temperatures.

The review is organized as follows. In section 1 the general model is presented. In section 2 the adiabatic approach is discussed. In section 3, the range of validity of the adiabatic approach is analyzed and, in section 4, the method is applied to the case of thermoelectric transport. In section 5, the results of the adiabatic approach in the Coulomb blockade regime are investigated. In section 6, the effects of time dependent perturbations are thoroughly studied.

## Review

### 1 Model

In this section, we present a general Hamiltonian for a multilevel quantum dot or molecule including the local coupling to vibrational degrees of freedom and connected to two leads in the presence of a finite bias voltage and temperature gradient. Contrary to previous reviews on the subject [78], we include the effect of time-dependent perturbations, such as an external antenna and pumping terms between the nanosystem and the leads.

The total Hamiltonian of the system is

[1]
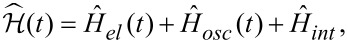


where 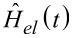 is the electronic Hamiltonian, 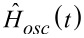 describes the vibrational degrees of freedom in both the leads and the dot, and 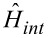 describes the interaction between the electronic and vibrational modes on the dot.

The electronic Hamiltonian 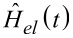 is given by

[2]
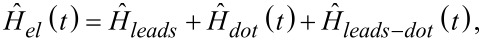


the different terms of which are introduced in the following. The dot Hamiltonian is

[3]
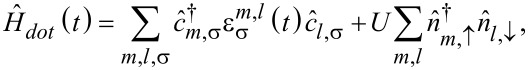


where *c_m_*_,σ_ (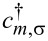) is the standard electron annihilation (creation) operator for electrons on the dot levels with spin σ = ↑, ↓, where the indices *m*,*l* can assume positive integer values with a maximum *M* indicating the total number of electronic levels in the quantum dot. The matrix 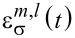 is assumed to be diagonal in spin space, while *U* represents the Coulomb repulsion between the electronic levels. We assume that only the diagonal part of the matrix 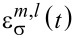 is non-zero and contains an assigned time dependence coming from the effects induced by an external antenna


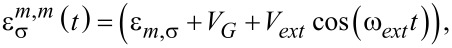


where *V_ext_* is the amplitude of the external antenna potential, ω*_ext_* is the driving frequency, *V_G_* is the static gate potential, *e* is the electron charge, and ε*_m_*_,σ_ are the bare energies of the quantum dot levels. It is important to notice here that in this review we will consider two possible ways in which the electromagnetic field generated by an external antenna couples to the dot degrees of freedom: One is described in Equation 3, where the external field excites directly the electronic dot levels (see section 6.2 and [79]); the second case will be introduced in the Hamiltonian in Equation 4 and involves the coupling of the field with the mechanical displacement of the dot (see section 6.1 and [77]).

The Hamiltonian of the leads is given by

[5]
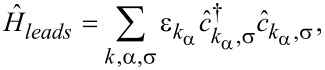


where the operators 
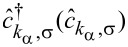
 create (annihilate) electrons with momentum *k*, spin σ, and energy 
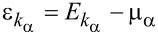
 in the left (α = *L*) or right (α = *R*) leads. The difference of the electronic chemical potentials in the leads provides the bias voltage *V_bias_* applied to the junction: μ*_L_* = μ + *eV_bias_*/2, μ*_R_* = μ − *eV_bias_*/2, with μ being the average chemical potential. The left and right leads will be considered as thermostats in equilibrium at the temperatures *T_L_* = *T* + Δ*T*/2 and *T_R_* = *T* − Δ*T*/2, with *T* being the average temperature. Therefore, the left and right electron leads are characterized by the free Fermi distribution functions *f_L_*(ω) and *f_R_*(ω), respectively.

The coupling between the dot and the leads is described by

[6]
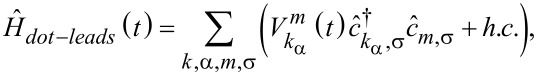


where the tunneling amplitude between the molecular dot and a state *k* in the lead α has in general a time-dependent amplitude 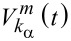. For the sake of simplicity, we will suppose that the density of states ρ*_k_*_,α_ for the leads is flat within the wide-band approximation: 
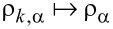
, 
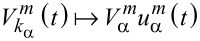
, with 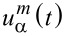 being periodic functions describing the strength of the pumping external parameters. Therefore, the time-dependent full hybridization width matrix of the molecular orbitals is 
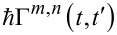
 = 
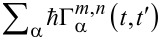
 = 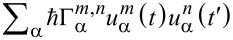, with the Planck constant 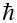 and the tunneling rate 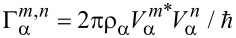. In this review, we consider the generic asymmetric configuration: 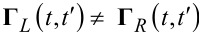, where bold letters indicate matrices.

The vibrational degrees of freedom in the system are described by the Hamiltonian

[4]
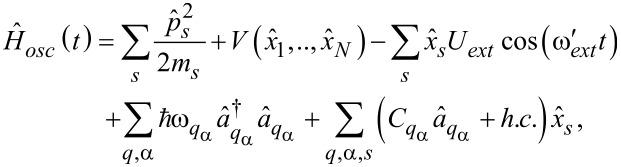


where *m_s_* is the effective mass associated with the *s*-th vibrational mode of the nanosystem, 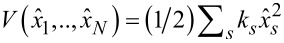 is the harmonic potential (with *k_s_* being the spring constants, and 
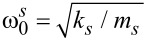
 being the oscillator frequencies), *U_ext_* is the force of the external antenna, 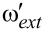 is the driving frequency and 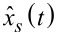 is the displacement field of the vibrational modes of the quantum dot.

In Equation 4, the operators 
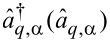
 create (annihilate) phonons with momentum *q* and frequency ω*_q_*_,α_ in the lead α. The left and right phonon leads will be considered as thermostats in equilibrium at the temperatures *T_L_* and *T_R_*, respectively, which we assume to be the same as those of the electron leads. In the following we will include also the presence of the phonon bath in the leads when we derive the equations relevant for the adiabatic approach. Their effect will be considered in section 4 where the thermoelectric properties of a molecular junction will be analyzed. In Equation 4, the coupling between the displacement 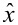 and a phonon *q* in the lead α is given by the elastic constant *C_q_*_,α_. In order to characterize this interaction, one introduces the spectral density *J*(ω):

[7]
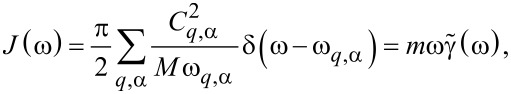


with *M* being the mass of the lead atoms and 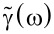 the frequency-dependent memory-friction kernel of the oscillator [80]. In the regime 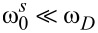 for all the modes, 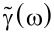 can be approximated as real and independent of frequency, providing the damping rate 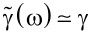. [80] If not specified, we consider the symmetric configuration: γ*_L_* = γ*_R_* = γ/2.

In this review, we assume that the electronic and vibrational degrees of freedom in the metallic leads are not interacting [2,81], in the sense that the electron–phonon coupling active in the leads gives rise to effects on the nanoscale which are negligible when compared with those due to the interaction between intra-dot or intra-molecular electronic and vibrational degrees of freedom. Therefore, the electron–vibration coupling is assumed effective only on the quantum dot. This coupling is assumed to be linear in the vibrational displacements and proportional to the molecule electron occupations

**Figure 1:**
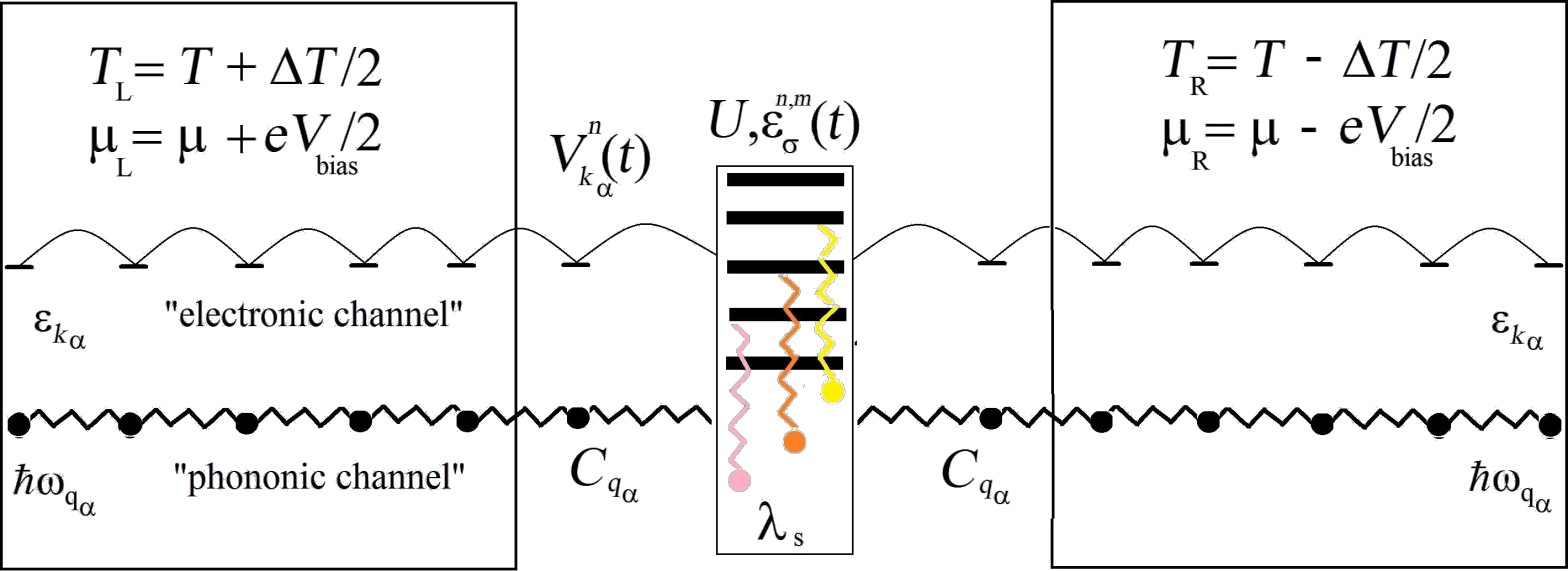
Scheme of the device studied in this work. The left lead (LL) and the right lead (RL) are kept at different chemical potentials μ*_L_* = μ + *eV_bias_*/2, μ*_R_* = μ − *eV_bias_*/2, and different temperatures *T_L_* = *T* + Δ*T*/2, *T_R_* = *T* − Δ*T*/2. The pumping signals are applied using the gates 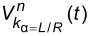, while the back gate (Gate) induces a shift of *V_G_* to the quantum dot energy levels 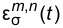 in the presence of a local Coulomb repulsion *U* and coupling λ*_s_* with *N* vibrational modes. The electronic channel in the leads is indicated by 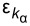, while the phonon one by the energies 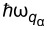.

[8]
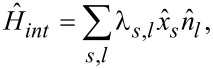


where *s* = (1,..,*N*) with *N* being the total number of vibrational modes, *l* = (1,..,*M*), 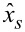 is the displacement operator of the *s* vibrational mode, 
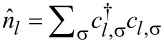
 is the electronic occupation operator, and λ*_s,l_* is a matrix representing the electron–vibrational coupling. A schematic representation of the device is presented in Figure 1.

### 2 Adiabatic approach

In this review, we consider the electronic system coupled to very slow vibrational modes and temporal perturbations: 
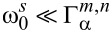
, 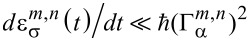, 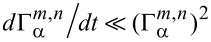, for each *s*, α and all pairs of (*m*,*n*). In this limit, we can treat the mechanical degrees of freedom as classical, acting as slow classical fields on the fast electronic dynamics. Therefore, the electronic dynamics is equivalent to a time-dependent multi-level problem with energy matrix 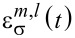 → 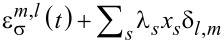, where *x_s_* are now classical displacements.

We point out that an extensive presentation of the adiabatic approximation for vibrational degrees of freedom in a quantum dot has already been presented in [78], but here we extend that approach to the case where a thermal gradient, phonon leads degrees of freedom, and time-dependent perturbations, such as external antenna and pumping terms, are present. The Langevin equation for the vibrational modes of the quantum dot (or molecule), including all the mentioned extensions, can be cast as follows

[9]
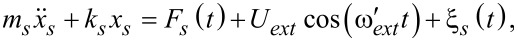


where the generalized force

[10]
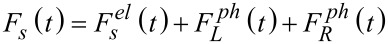


contains the contribution 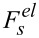 due to the effect of all electronic degrees of freedom, and 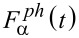 is the force due to the coupling to the α lead phonon degrees of freedom. It can be easily shown that, in the regime investigated in this review, in Equation 10, one has 
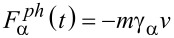
, with γ_α_ = γ/2.

The electronic force, *F^el^*(*t*) = tr[*i*λ*_s_****G****^<^*(*t*,*t*′)] (the trace “tr” is taken over the dot levels), is defined in terms of the lesser dot matrix Green’s function ***G****^<^*(*t*,*t*′), while the fluctuating forces ξ*_s_*(*t*) will be discussed later in this section.

For the sake of simplicity, we do not include explicitly the effect of the Coulomb repulsion on the quantum dot Hamiltonian in deriving equations encoding the adiabatic approximation. In section 5, we will show that, in the particular case of a single-level quantum dot with large repulsion *U*, the adiabatic approach works exactly as in the non-interacting case with the “caveat” of treating each Green’s function pole as a non-interacting level [82].

Our notation is such that G denotes full Green’s functions, while 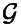 denotes the strictly adiabatic (or frozen) Green’s functions that are evaluated for a fixed value of ***X*** ≡ *x_s_*(*t*) and *t*. Starting from the Dyson equation

[11]
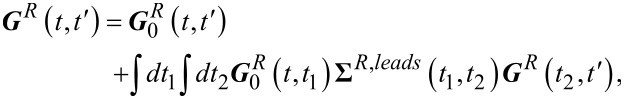


where 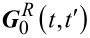 is the retarded Green’s function in the absence of coupling to the leads, it is straightforward to show that the adiabatic expansion (to first order in 
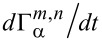
, 
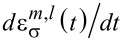
 and 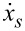) for the retarded Green’s function ***G****^R^* is given by

[12]
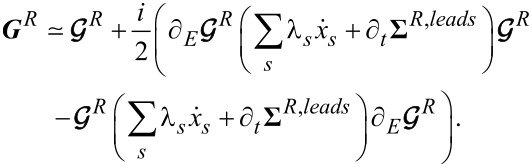


Above, 
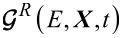
 is the strictly adiabatic (frozen) retarded Green’s function including the coupling with the leads

[13]
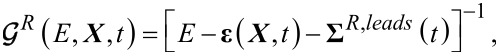


where 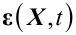 represents the matrix 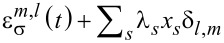 and 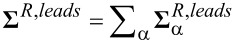 is the total self-energy due to the coupling between the dot and the leads. The self-energies 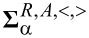 are defined as

[14]
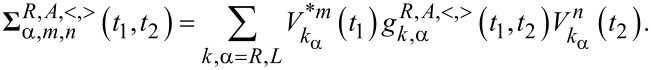


Notice that the imaginary part of 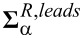 is proportional to 
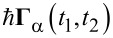
. Employing the above equations, for the lesser Green’s function ***G****^<^* one gets

[15]
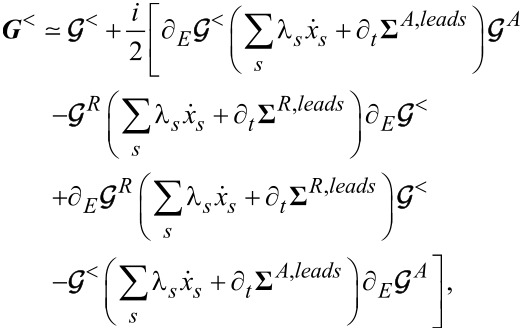


with 
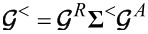
.

The electron-vibration-induced forces in the zero-th order adiabatic limit, 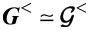, are given by

[16]
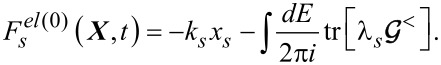


The leading-order correction to the lesser Green’s function ***G****^<^* gives two contributions to the electron-vibration induced forces: a term proportional to the vibrational velocity and a pumping velocity term acting as a driving force

[17]
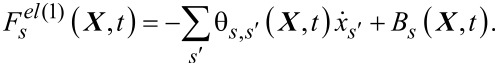


The first term determines the tensor **θ** (obtained after integration by parts)

[18]
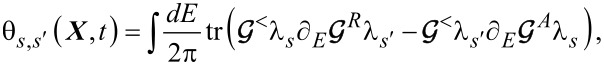


while the second term determines the vector *B*

[19]
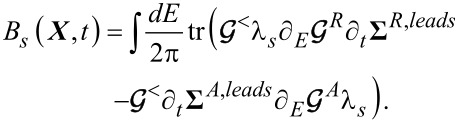


The tensor **θ** can be split into symmetric and anti-symmetric contributions [83], **θ = θ***^sym^* + **θ***^a^*, which define a dissipative term **θ***^sym^* and an orbital, effective magnetic field **θ***^a^* in the space of the vibrational modes. The latter interpretation is based on the fact that the corresponding force takes a Lorentz-like form. Using 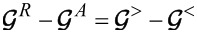 and noting that 
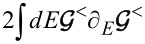
 = 
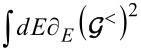
 = 0, we obtain the explicit expressions

[20]
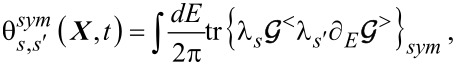


[21]
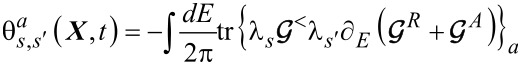


Here we have introduced the notation 
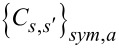
 = 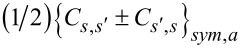 for symmetric and anti-symmetric parts of an arbitrary matrix C.

We can now discuss the stochastic forces ξ*_s_*(*t*) in Equation 9 in the adiabatic approximation. In the presence of coupling with the phonon leads, the fluctuating forces are composed of three independent terms

[22]
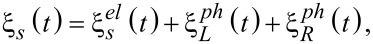


where 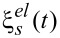 represents quantum-electronic fluctuations while 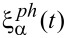 is due to the α phonon lead. The noise term 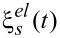 expresses the effects of the quantum-electronic density fluctuations 
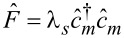
 on the oscillator motion. By analogy with fluid dynamics, it can be viewed as originating from the thermal and non-equilibrium fluctuations of the electronic “fluid” in which the vibrating quantum dot can be considered as immersed. In the absence of electron–electron interactions, the Wick theorem allows one to write the noise correlator as

[23]
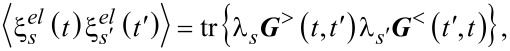


where G*^>^*(*t*,*t*′) is the greater Green’s function with matrix elements

[24]
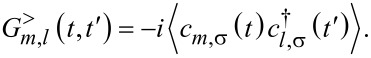


At this stage, it represents a colored-noise term in the Langevin equations (Equation 9) that depends non-locally on the dynamics of the vibrational modes and it is complicated to treat numerically. In the adiabatic approximation, one first substitutes the full Green’s function G by the adiabatic zero-order Green’s function 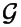 and then observes that the electronic fluctuations act on short time scales only. For this reason the total forces ξ*_s_*(*t*) are locally correlated in time and one has to only retain the low-frequency limit of their stochastic variance. One thus obtains a multiplicative white-noise term

[25]
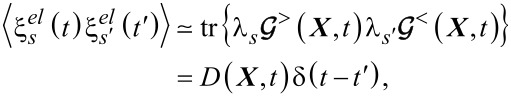


where

[26]
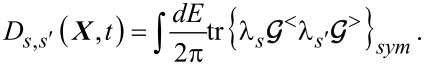


The fluctuating forces coming from the fluctuations of the phonon leads 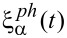 have the following property


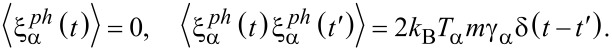


Combining the three terms, one gets the total fluctuating forces ξ*_s_*(*t*) such that

[27]
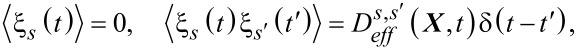


where the effective position-dependent noise term 
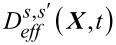
 is

[28]
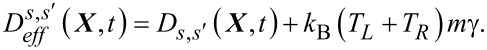


Once the forces in Equation 16 and Equation 17 and the noise terms in Equation 25 are calculated, Equation 9 represents a set of nonlinear Langevin equations in the unknown dynamics *x_s_*(*t*), which are explicitly dependent on time. Even for the simple case where only one vibrational degree of freedom is present, the stochastic differential equation should be solved numerically. In [76], the present authors have applied a Runge–Kutta algorithm adapted to the stochastic nature of the equation. When an explicit time-dependence is present, such as in the case where the charge pumping is studied [79,84], the periodic nature of the temporal perturbations allows one to extend the algorithm straightforwardly. Indeed, by sampling the occurrences of ***X***,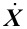 in the phase space at times *t* separated by the characteristic period of the perturbations, one can calculate the oscillator distribution functions *P*(***X***,***V***,*t*) (where 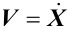) during a single period, and, therefore, all the properties of the vibrational modes. Using this function, one can determine the time evolution of an electronic or vibrational observable *O*(***X***,***V***,t*)* as:

[29]
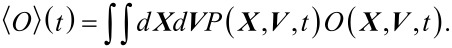


The electronic observables, such as charge and heat currents, can be evaluated exploiting the slowness of the vibrational degrees of freedom.

#### 2.1 Electronic charge and heat currents

In this subsection, we discuss the adiabatic expansion for the current passing through the quantum dot. An expression valid in the absence of time-dependent perturbations it has already been provided in [83], but here we re-present that derivation in the presence of time-dependent perturbations. The definition of the current through lead α is given by

[30]
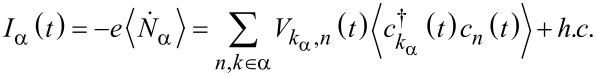


where 
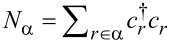
. Using the definitions of self-energy and Green’s functions [81] the current can be expressed in terms of the Green’s function of the dot and the self-energy of the dot–leads coupling

[31]
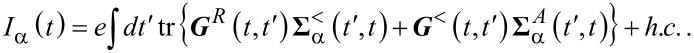


We can now apply the adiabatic expansion to the above expression employing the formulas for the Green’s functions ***G****^R^* (Equation 12) and ***G****^<^* (Equation 15)

[32]
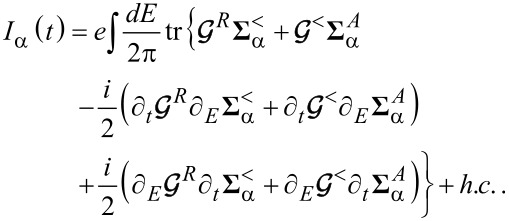


We split the current into an adiabatic contribution *I*_0_, two terms proportional to the velocity 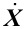 and the time derivative of the pumping parameters *u*_α_(*t*) (describing the lead–dot self-energy **Σ**(*t*): 
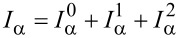
. The zero-th order adiabatic contribution is given by

[33]
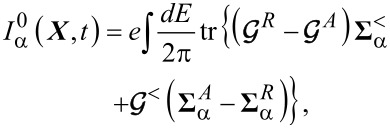


where we have collected the strictly adiabatic terms from Equation 12 and Equation 15. Now we turn to the first-order corrections, restricting our considerations to the wide-band limit. The contribution to the current (Equation 32), which is linear in the velocity of the vibrational modes, reads (after integration by parts)

[34]
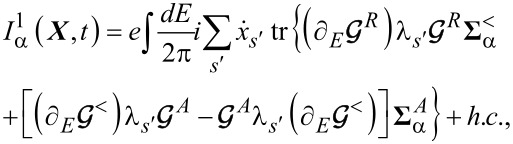


while the term 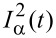 coming from the pumping perturbations is

[35]
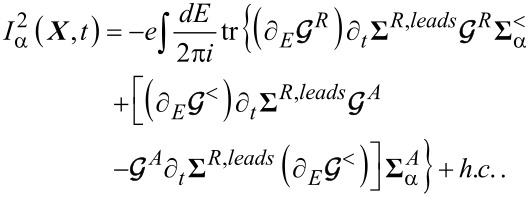


Analogously to Equation 31, the electronic energy current passing through the dot from the lead α = *R*,*L* is defined as 
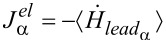
. In the case where the chemical potentials of the leads are not time-dependent, one can easily show that all the expressions derived for the electronic current *I*_α_ and its adiabatic expansion are formally identical to those valid for the energy current 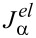, with the only “caveat” that one has to substitute the self-energies 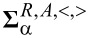 with the functions 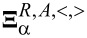 defined as

[36]
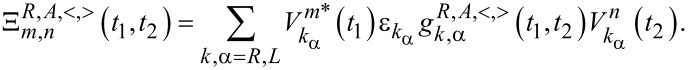


In analogy with the terms in the deterministic and fluctuating forces of the Langevin equations (Equation 9), the total energy current *J* involving the oscillator is composed of three terms [72]:

[37]
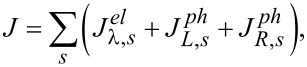


where 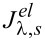 originates from the electron level and depends on the electron–vibration coupling. It can be also expressed as

[38]
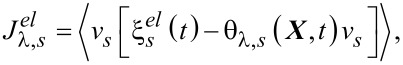


while 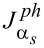 comes from the α phonon lead

[39]
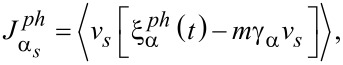


where 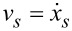. These quantities have to be evaluated along the dynamics. Once the stationary state is reached, the energy conservation requires that the total energy current *J* vanishes.

In the next section we discuss the validity of the adiabatic approximation, that is based on the separation between the slow vibrational and fast electronic timescales.

### 3 Range of validity of adiabatic approach: single-level molecule

In this section, we investigate the range of validity of the adiabatic approach together with the semiclassical treatment of the vibrational degrees of freedom. To this aim, we consider, as in the rest of the paper, the simple case where the nanoscopic system is represented by a molecule modeled as a single electronic level (*M* = 1) locally interacting with a single vibrational mode (*N* = 1, see Figure 1). This means that the focus is on a molecular (or quantum dot) electronic orbital that is sufficiently separated in energy from other orbitals. We have in mind, for instance, the C_60_ molecule when the LUMO energy differs from the HOMO energy for more than 1 eV. Even when the degeneracy of the LUMO is removed by the contact with Ag leads, the splitting gives rise to levels that are separated by an energy of the order of 0.5 eV [85]. Furthermore, the energy of the molecular orbital can be tuned by varying the gate voltage *V_G_* and unless otherwise stated we do not consider time-dependent perturbations.

We will focus on the center-of-mass mode as the only relevant vibrational mode for the molecule, which is expected to have the lowest frequency for large molecules. In fact, in C_60_ molecules, experimental results provide compelling evidence for a coupling between electron dynamics and the center-of-mass motion where 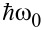 has been estimated to be of the order of 5 meV [9]. Furthermore, as reported in experimental measurements [9], the effects of the electron–vibration interaction are not negligible in junctions with C_60_ molecules. Within these assumptions, in Equation 1, the interaction Hamiltonian 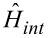 reduces to the same interaction term of the single impurity Anderson–Holstein model [2] and the dot–oscillator coupling sets the characteristic polaron energy and length scales

[40]
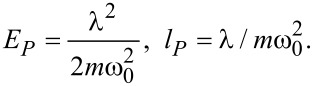


Equation 9 reduces in this case to a single Langevin equation [76,86]. Since the main objective of this section is to discuss the range of validity of the adiabatic approach for the electronic properties, we hereby report the expression for the displacement-dependent electronic spectral function *A*(ω,*x*)

[41]
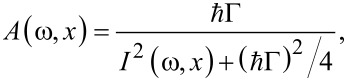


with 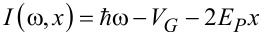. Within the adiabatic approach, the actual electronic spectral function *A*(ω) is

[42]
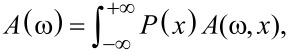


where *P*(*x*) is the reduced position-distribution function of the oscillator in the absence of time-dependent forces. Another important quantity is the average kinetic energy of the oscillator

[43]
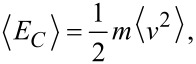


which is calculated through the reduced velocity distribution function *P*(*v*) of the oscillator in the absence of time-dependent forces.

#### 3.1 Equilibrium conditions at *T* ≠ 0

In this subsection, we consider equilibrium conditions with both leads at temperature *T*. When the gate voltage is tuned in such a way that the electronic level of the dot is far from the bias window (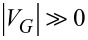), a very low electronic density is on the dot. We emphasize that, in this limit, the electronic Green’s functions of the model can be exactly calculated if, as assumed in this revew, the wide band limit is used for the leads [87–88]. We compare an approach that takes fully into account the quantum nature of the oscillator, and it is valid also at very low temperatures (*T* ≤ ω_0_), with the adiabatic approach. We stress that, within the off-resonant regime, the oscillator dynamics is very weakly perturbed by the effects of the electron–vibration coupling, but it remains sensitive to the coupling to phonon leads.

The spectral function calculated within the exact approach in the regime of low level occupation has been compared with that obtained within the adiabatic approach. We focus on the electronic spectral function *A*(ω) since in the linear response regime (low bias voltage, absence of time perturbations) all the transport coefficients can be expressed as integrals of *A*(ω) (see next subsections and the remaining sections of the present paper). It is clear that a good agreement between the adiabatic approach and the exact approach in the low density regime can be used as a reliable test of the validity of the adiabatic scheme. The comparison between the two approaches represents a new and interesting part of the review and it will allow us to assess that quantities calculated within the adiabatic approach are very reliable even in the regime of higher density.

**Figure 2:**
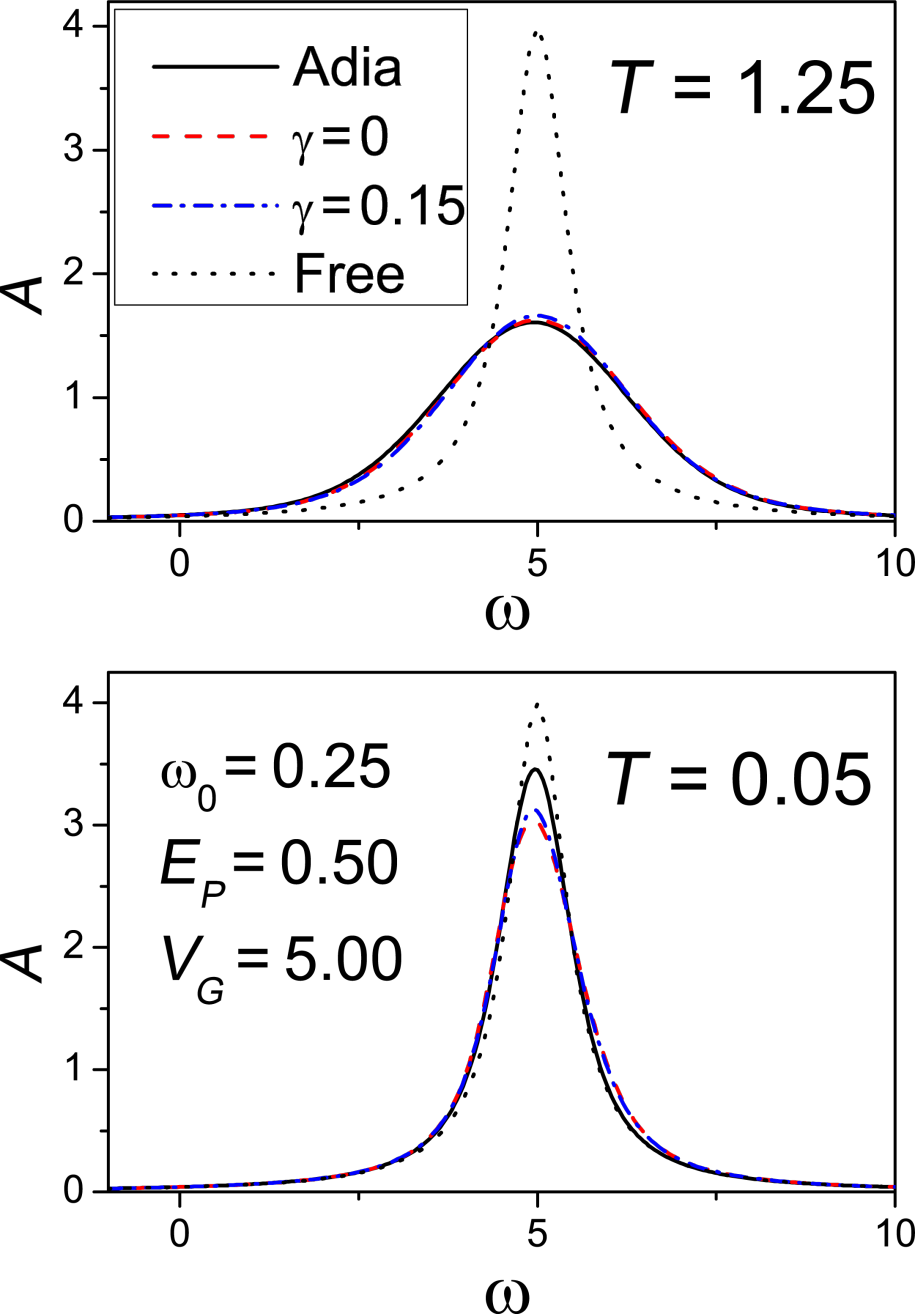
Analysis of the validity of the adiabatic approximation. Electronic spectral function as a function of the frequency (in units of Γ) for adiabatic approach (solid line), fully quantum low-density approach (dashed line for γ = 0, dash-dotted line for γ = 0.15), and free case (dotted line) at temperature *T* = 1.25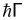/*k*_B_ (upper panel) and temperature *T* = 0.05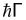/*k*_B_ (lower panel). In all the plots, *E_P_* = 0.5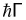, *V_G_* = 5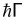, and ω_0_ = 0.25Γ.

In the upper panel of Figure 2, we consider the spectral functions at *T* = 1.25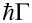/*k*_B_, which is close to room temperature for 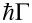 ≈ 20 meV. Moreover, we consider the off-resonant regime *V_G_* = 5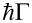. The spectral weight up to 0 (position of the chemical potential) indicates that the level occupation *n* is small (less than 0.1). The agreement between the spectral functions calculated within the two approaches is excellent. The peak positions for both approaches are at ω = *V_G_* and the widths of the curves match perfectly. The role of γ is not relevant, since, in any case, it is much smaller than Γ. Obviously, with decreasing the temperature, the two approaches tend to differ. In the lower panel of Figure 2, we have considered a very low temperature (*T* = 0.05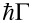/*k*_B_) where a worse agreement is expected. We point out that the agreement between the two approaches is still good. Actually, the exact approach at low molecule occupation slightly favors a small transfer of spectral weight at high frequency. In any case, the strong similarities in the spectral function will point out to analogous behaviours of electron transport properties within the two approaches. In conclusion, we can say that the semiclassical adiabatic approach correctly describes the system down to quite low temperatures.

In the linear response regime, without time perturbations, the spectral function allows one to derive all the Green’s functions using the fluctuation–dissipation theorem. Clearly, out of equilibrium (finite bias and/or time perturbations) not only the imaginary part of the retarded Green’s function is important, but, as discussed in the previous section, also other Green’s functions are relevant. In this review, we will analyze the effects of non-equilibrium Green’s functions directly on the out-of-equilibrium response functions (for example, currents and pumping charges). It is well known that, in the out-of-equilibrium regime, the self-consistent adiabatic approach becomes progressively more exact in comparison with the equilibrium and linear response regime. For example, in the next subsection, we will show that the bias voltage introduces an additional effective temperature for the vibrational degrees of freedom, therefore the semiclassical approach represents more and more the physical situation.

#### 3.2 Non-equilibrium conditions at *T* = 0

**Figure 3:**
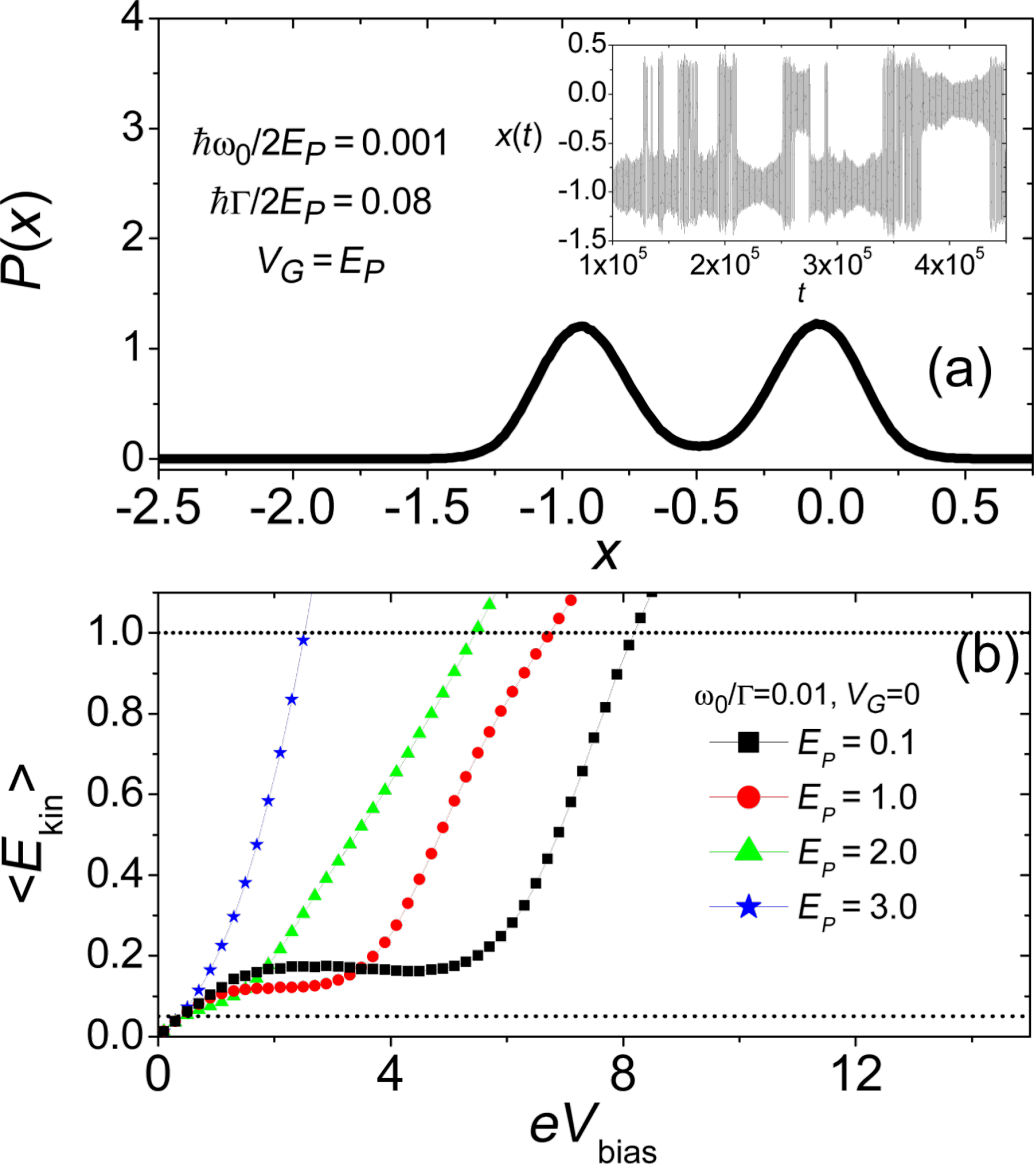
Analysis of the validity of the adiabatic approximation. Panel (a): Dimensionless position distribution probability for *eV_bias_*/2*E_P_* = 0.1. Inset of panel (a): Solution of the Langevin Equation (Equation 27) for the same values of parameters as in the main plot. Panel (b): Average kinetic energy 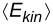 as function of the bias voltage for different interaction strengths *E_P_*. Adapted from [76].

**Figure 4:**
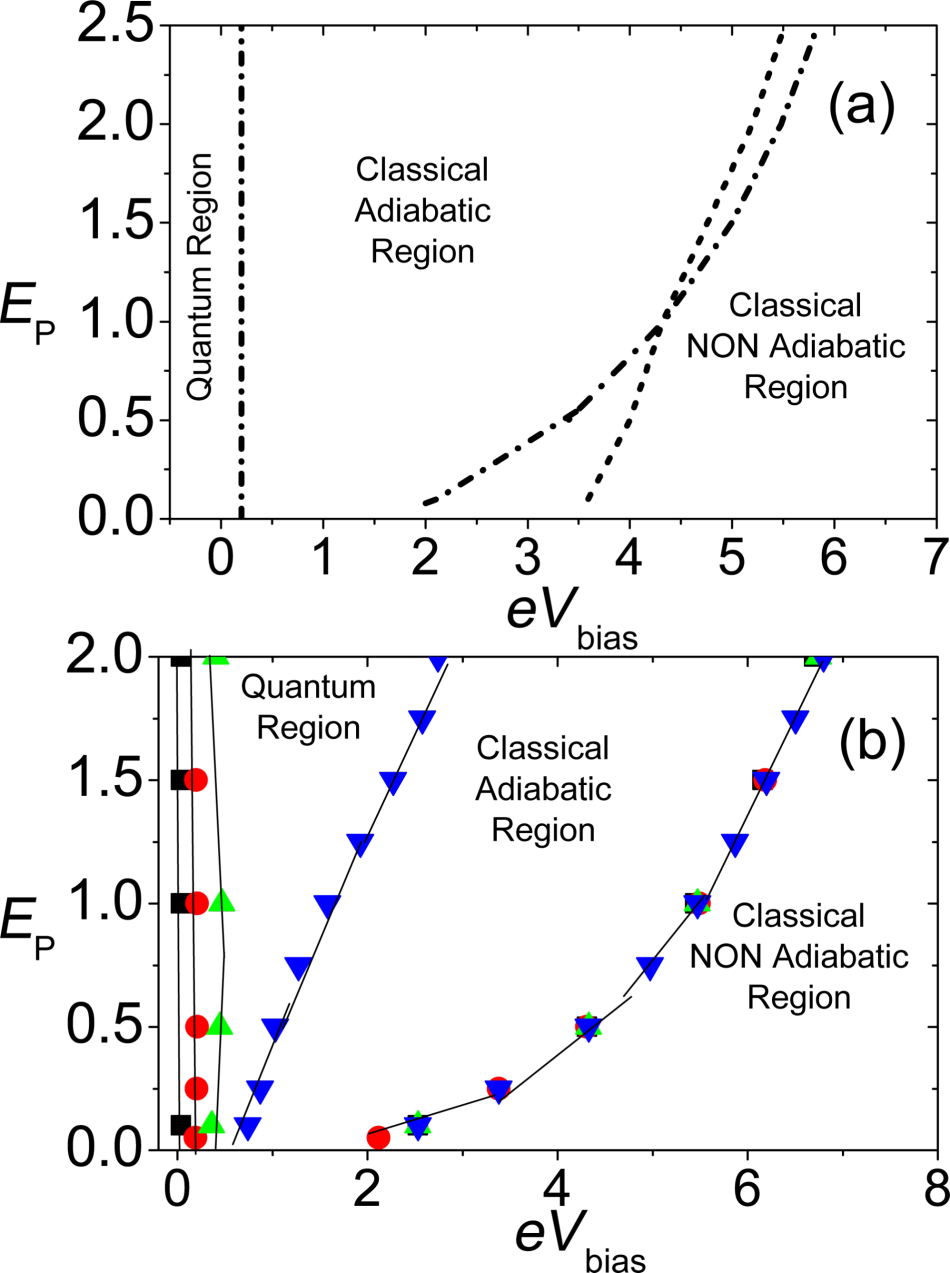
Phase diagrams expressing the validity of the adiabatic approximation. Panel (a): Phase diagram at fixed adiabatic ratio ω_0_/Γ = 0.05. The dashed (black) line indicates the QR–CAR crossover for *V_G_* = 0 and *V_G_* = 1. The dotted and dashed dotted lines indicate the CAR–CNAR crossover for *V_G_* = 0 and *V_G_* = 1, respectively. Panel (b): Phase diagram at fixed gate voltage *V_G_* = 0 (asymmetric static potential) for different adiabatic ratios ω_0_/Γ = 0.01, 0.05, 0.1 and 0.25. Adapted from [76].

In this subsection, we consider γ = 0 (no coupling to the lead-phonon degrees of freedom) and no temperature gradient between the electronic leads, which are considered to be at zero temperature *T_L_* = *T_R_* = *T* = 0. A careful analysis of this regime has been provided by the present authors in [76]. Here, we review the main results of that analysis for the sake of completeness. The first step of that analysis consists in obtaining the displacement distribution probability, *P*(*x*), associated to the dynamics of the nanosystem vibrational mode. It results from the solution of a single Langevin equation with no forcing term, see Equation 9 and [76]. In Figure 3a, *P*(*x*) is shown in the strong-coupling regime 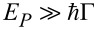 at equilibrium for zero temperature. We highlight here that the bi-modality is provided by the interaction between the electronic and vibrational degrees of freedom which modifies the generalized force in Equation 10. The actual dynamics of the position of the oscillator *x*(*t*) is plotted in the inset of the same panel in Figure 3 and shows the “jumps” between the two symmetric potential wells. The kinetic energy of the oscillator provides a useful quantity for checking the validity of the adiabatic approximation. In Figure 3b, it is plotted as a function of the bias voltage applied to the junction. As one increases the electron–oscillator interaction, one can see that a non-monotonic region at intermediate bias voltages appears. Interestingly, for small bias voltages, all the curves show a linear behaviour (not shown), meaning that in this regime the non-equilibrium electronic bath provides an effective temperature proportional to the bias *eV_bias_*. As already stated in [76], by using the kinetic energy of the oscillator one can build a phase diagram of the model defining the range of validity of the adiabatic approximation. By comparing the average kinetic energy with the vibrational energy 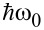 and the characteristic electronic energy 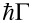, one can define a quantum region (QR) (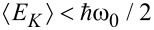), a classical adiabatic region (CAR) (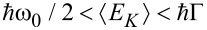), and finally a classical non-adiabatic region in the phase diagram (CNAR) (
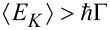
). The analysis is reported in Figure 4. We expect that the adiabatic approximation is reliable in the intermediate classical adiabatic region that occupies the majority of the area shown in Figure 4a, where the phase diagram in the plane (*V_G_*, *eV_bias_*) is shown. The quantum region is confined in a striped area on the left of the plot corresponding to small values of the bias voltages, as physically expected. The boundary between the CAR and the CNAR regions is slightly dependent on the gate voltage, showing that in the regime of low dot-density occupation the validity of the approach is stronger. The plot reported in Figure 4b shows the phase diagram in the same plane as in panel (a) for different values of the vibrational energy 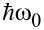, showing that the boundary QR–CAR moves to larger values of the bias voltage while the CAR–CNAR boundary is almost unaffected. As a consistency check of the adopted approach we observe that, if one increases the characteristic energy of the vibrational mode, the area of validity of the semiclassical adiabatic approximation shrinks.

### 4 Charge and heat transport

In this section we analyze the electronic transport properties of our quantum dot (or molecule) in the regime of validity of the adiabatic approach. In particular, we will focus on electronic properties as electrical and thermal conductivities resulting from the average over the dynamical fluctuations of the oscillator motion. To this aim, we report the zero-th order adiabatic expression for the electronic current *I*^(0)^ (averaged over the distribution probability of the oscillator *P*(*x*,*v*))

[44]
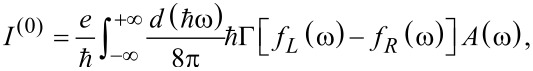


and the conductance *G*

[45]
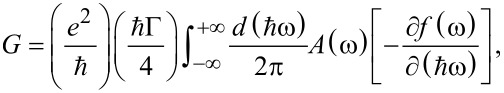


where *A*(ω) is the spectral function defined in Equation 42, with 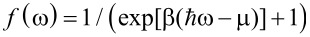 the free Fermi distribution corresponding to the chemical potential μ and the temperature *T*, and β = 1/*k*_B_*T*.

**Figure 5:**
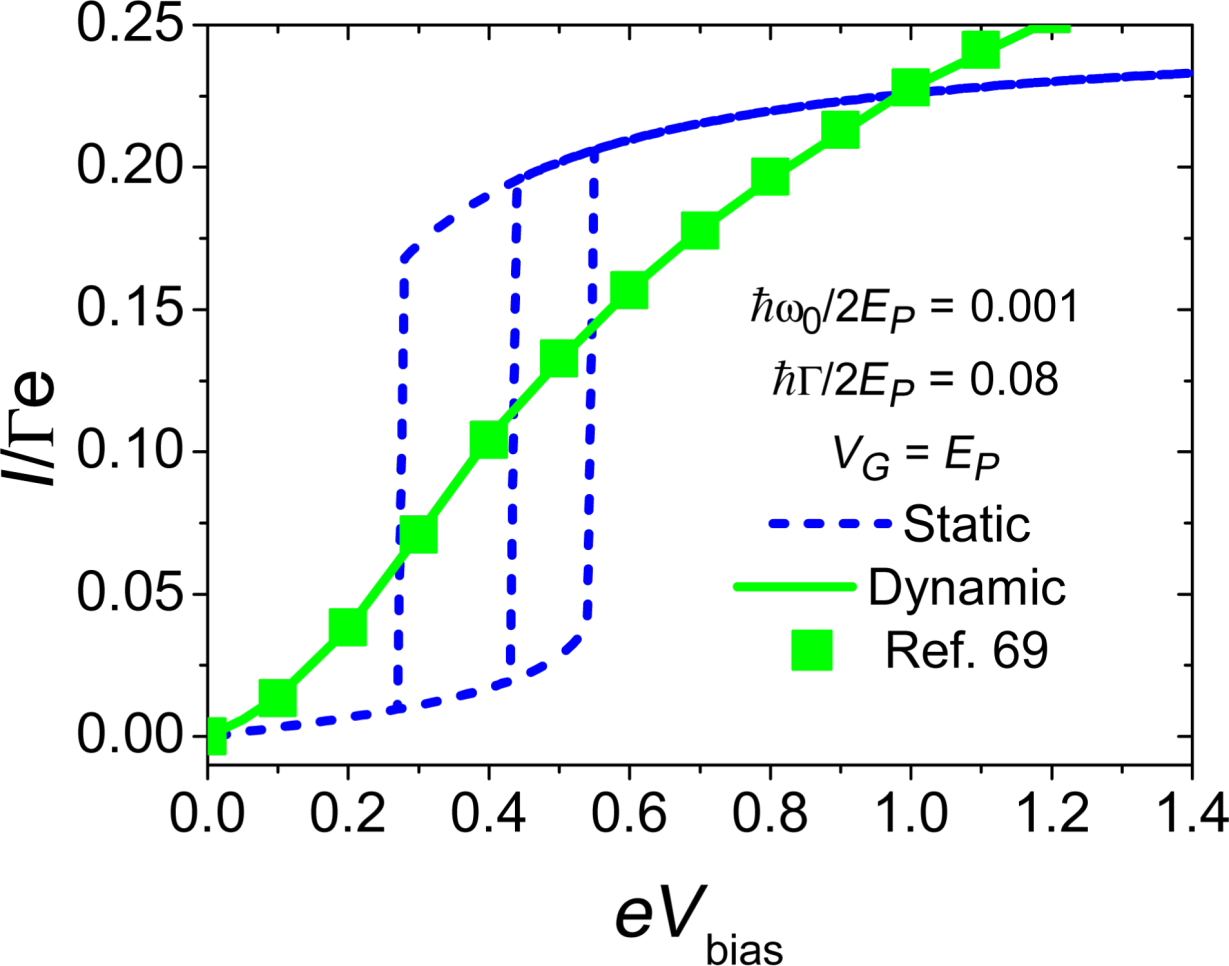
Electronic transport properties within the range of validity of the adiabatic approximation. Current (*e*Γ units) voltage (*eV_bias_* in units of 2*E_P_*) characteristic of the device. Solid (green) curve is drawn from [69], squares indicate the results of the semiclassical adiabatic approach adopted in this paper and dashed (blue) line indicates the *I*–*V_bias_* in the infinite mass static approximation. Adapted from [76].

We can now discuss briefly one of the main results for the current–voltage characteristic given by the adiabatic approach at zero temperature. In Figure 5, the *I*–*V_bias_* characteristic for *V_G_* = *E_P_* = 2 is shown comparing the adiabatic approach adopted in this paper (squares), with the limit where the mass of the oscillator is considered as infinitely large (static approximation) and no dynamics is calculated (dashed line). Furthermore, we report, as a full line (green on line), data provided by an independent calculation in [69], which agree completely with our calculations. As one can see, the results obtained in the adiabatic limit completely wash out the hysteresis effects and bi-stability that is obtained in the static approximation, showing that the inclusion of the slow dynamics of the oscillator structurally modifies the *I*–*V_bias_* curve. A similar scenario is obtained for the electronic conductance (not shown in Figure 5). In this case, the dynamical correction due to the adiabatic approach gives a substantial broadening of the resonance peak that is shifted by the polaronic effect [76].

We devote the rest of this section to the discussion of the thermoelectrical properties of a molecular junction in the linear-response regime. Our focus will be manly on the role of the electron–vibration coupling. To this aim, we introduce a temperature gradient between the leads, and focus on the linear-response regime around the average chemical potential μ and the temperature *T* (Δ*T* → 0, *V_bias_* → 0). We also introduce the possibility of the interaction between the relevant vibrational mode of the molecule and the phonons in the leads. Indeed, if this mode is elastically coupled with a neighbour atom of the leads by a spring with constant *k*′, one gets for the dissipative contribution of the leads in the dynamics of the mode 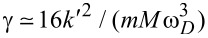. As in the previous section, we consider parameters appropriate for molecular junctions based on C_60_ molecules. Taking the mass *m* of the C_60_ molecule and the atomic mass *M* of Ag, Au, and Pt (typical metallic leads), 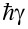 is of the order of 7.68, 7.74, and 2.98 meV, respectively. The smallest value of coupling to phonon baths is due to the largest Debye frequency ω*_D_* of platinum. In any case, 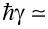 3–8 meV for these metals, therefore ω_0_ is of the same order of γ.

Within the general approach discussed in the previous sections, we can calculate all the observable quantities relevant for studying the thermoelectric properties. For example, the Seebeck coefficient is given by *S* = *−G_S_*/*G*, where the charge conductance *G* has been defined in Equation 45, and

[46]
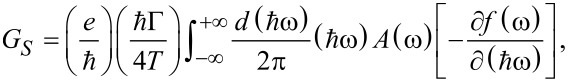


with *f*(ω) the free Fermi distribution. Then, we will calculate the electron thermal conductance 
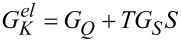
, with

[47]
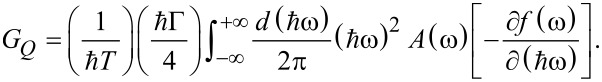


In order to estimate the thermal conductance, one can determine the vibrational energy currents directly from the derivative of the oscillator energy [89]. The oscillator is directly in contact with phonon leads, but only indirectly with electron leads due to the coupling between the oscillator and the electronic level on the molecule (see Figure 1). The phonon thermal conductance 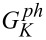 can be calculated within the linear-response regime [90–91] around the temperature *T* as

[48]
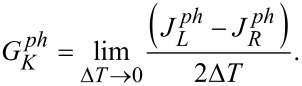


The total thermal conductance is then given by the sum *G_K_* of the electron and phonon thermal conductance:

[49]
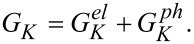


Therefore, one can easily evaluate the total figure of merit *ZT* (valid in the linear response regime):

[50]
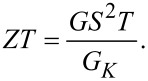


When the coupling of the center of mass mode to the metallic leads can be neglected (γ = 0), 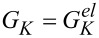, so that *ZT* = *ZT^el^*, which characterizes the electronic thermoelectric properties. When γ is different from zero, the contribution of the thermal phonon conductance 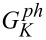 is not negligible in the determination of the figure of merit *ZT*. Not only the electronic transport functions but, as discussed below, also 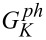 are sensitive to the effects of the electron–vibration coupling and tend to reduce the value of *ZT*.

In this section, we assume 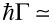 20 meV as energy unit. As a consequence, Γ will be the frequency unit. We will also assume ω_0_ = 0.25Γ and vary γ from 0.15Γ to 0.40Γ (simulating, as discussed above, the effects of different metallic leads). We will measure times in units of 1/Γ, temperatures in units of 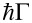/*k*_B_ (ambient temperature 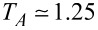 in these units). Finally, we fix the average chemical potential at μ = 0. Our analysis will mainly focus on the transmission of phonon energy and on the variation of the electronic level with respect to the chemical potential of the leads. These variations can be controlled, in our model, changing the gate potential *V_G_* from *V_G_* = 0, when the electronic level coincides with the lead chemical potential (resonant case), to a very different value 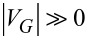 (off-resonant case).

In order to investigate heat exchange with the molecule, the leads phonon spectrum is assumed to be acoustic. For silver (atomic number *Z* = 47), gold (*Z* = 79), and platinum (*Z* = 78) leads considered in experimental measurements [92], the Debye frequency is such that 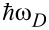 is of the order of 18.5, 15.1, and 20.7 meV, respectively [93]. Therefore, 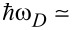 15–20 meV for these leads. In any case, as for any large molecule, the center of mass mode is such that 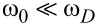.

**Figure 6:**
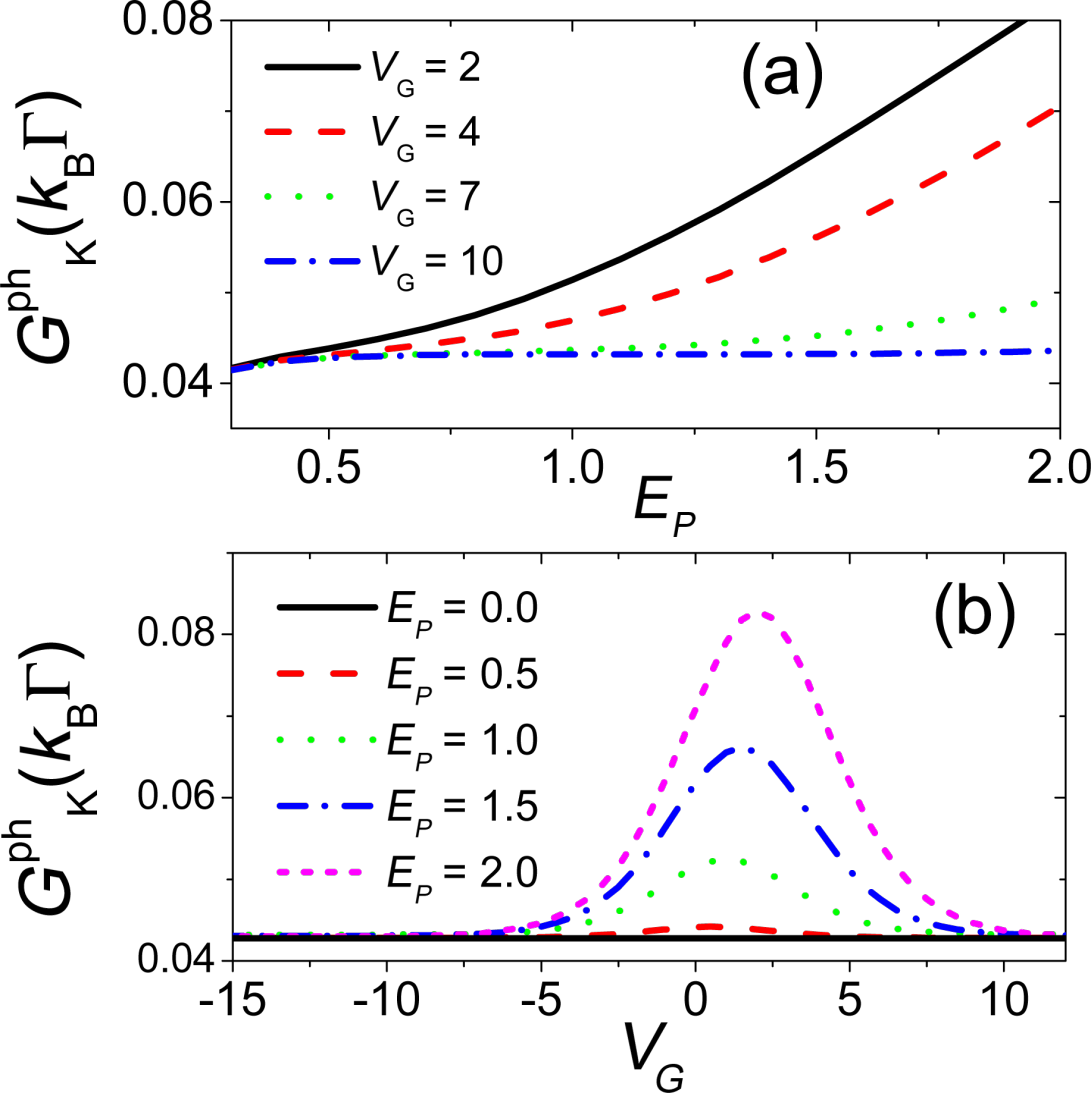
Phonon thermal conductance in the linear response regime, in the range of validity of the adiabatic approximation. Panel (a): Phonon thermal conductance 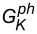 (in units of *k*_B_Γ) as a function of electron–vibration coupling *E_P_* (in units of 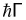) for different values of gate voltage *V_G_* (in units of 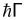). Panel (b): Phonon thermal conductance 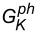 (in units of *k*_B_Γ) as a function of the gate voltage *V_G_* (in units of 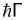) for different values of electron–vibration coupling *E_P_* (in units of 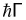). In all the panels, the oscillator damping rate γ = 0.15Γ, *T* = 1.25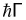/*k*_B_ (close to room temperature), and ω_0_ = 0.25Γ. Adapted from [86].

In Figure 6, we focus on the phonon thermal conductance 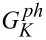. At moderate values of the coupling between the molecular oscillator and the lead phonon bath (
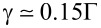
), we find that, for weak electron–vibration coupling *E_P_* (Figure 6a) or in the off-resonant regime 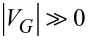 (Figure 6b), 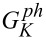 reaches its lowest value that is close to 0.04*k*_B_Γ 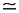 16 pW/K. One obtains this numerical value when electron–vibration effects are neglected (in the off-resonant regime, the electron level density is so low that the effective electron–vibration coupling is negligible). This value corresponds only to the contribution given by the phonon leads. We point out that this asymptotic value of 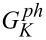 is always larger than the values of 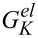 corresponding to *E_P_* = 0. Therefore, 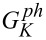 plays a primary role in determining the total thermal conductance *G_K_* for weak electron–vibration coupling. If one considers larger values of γ (for example γ 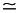 0.4Γ), 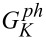 plays an even more important role in *G_K_*.

In Figure 6b, we show that 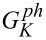 always gets larger with increasing the electron–vibration coupling *E_P_*. Actually, the electron–oscillator coupling gives rise to an additional damping rate on the vibrational dynamics whose effect is to enhance the thermal conductivity 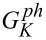. In a certain sense, due to the electron–vibration coupling, the molecular vibrational degrees of freedom are more effectively coupled to the lead phonons favouring the heat exchange between them. In the quasi-resonant regime (small *V_G_*), the increase of 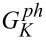 can be also favoured by the softening of oscillator frequency [77] due to the enhanced effects of electron–vibration coupling.

As discussed above, the effects of the electron–vibration coupling on the oscillator dynamics depends not only on the strength of the coupling *E_P_*, but also on the occupation of the electronic level. Actually, the behaviour of 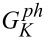 is strongly dependent on the value of gate voltage *V_G_*. As shown in Figure 6a, in the quasi-resonant case (*V_G_* = 2), the increase of 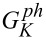 as a function of the electron–vibration coupling *E_P_* is marked. Actually, for *E_P_* = 2, the value of 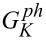 is doubled. On the other hand, in the off-resonant regime of low-level occupation, the dynamics of the oscillator is poorly influenced by the electron–vibration effects, even if *E_P_* is not small. Finally, in Figure 6b, we have analyzed the behaviour of 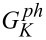 as a function of the gate voltage *V_G_* for different values of *E_P_*. As expected, 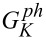 shows the largest deviations from the asymptotic value in the quasi-resonant case. We point out that the peak value is practically coincident with the value of *E_P_*, therefore, 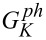 is strongly sensitive to the renormalizations of the electron level induced by the electron–vibration coupling. Indeed, the role of the phonon thermal conductance 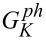 is important in inducing a suppression of *ZT*, which will be discussed in the next section.

### 5 Coulomb-blockade regime

In molecular junctions and quantum dots, the strong Coulomb repulsion usually reduces the electronic charge fluctuations and suppresses the double occupation of the electronic levels. These phenomena are known as Coulomb-blockade effects. In order to include this effect in the adiabatic approach discussed in the previous sections, we generalize it to the case in which the electronic level can be double occupied and a strong finite local Coulomb repulsion *U* is added together with the electron–vibration interaction.

The starting point is the observation that, in the absence of electron–oscillator interaction, and in the limit where the coupling of the dot to the leads is small 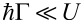 (first correction in 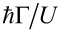 upon the atomic limit, see section 12.11 of [81]), the single-particle electronic spectral function on the dot is characterized by two spectral peaks separated by an energy interval equal to *U*. The peak at 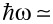 −*U* describes the single occupied electronic level, while the peak at 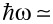 0 the doubly occupied one.

In the previous sections, we have seen that, one of the main effects of an adiabatic oscillator on the spectral peak at finite temperature is to give an extra broadening and a shift proportional to the oscillator–oscillator coupling energy *E_P_*. We therefore expect that, in the presence of an adiabatic oscillator 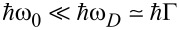 ≤ *k*_B_*T* one can perturb each spectral peak of the quantum dot independently, obtaining (see details in [82]) at the zero-th order of the adiabatic approach

[51]
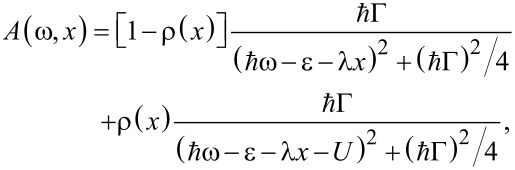


where ρ(*x*) is the electronic-level density per spin. In our computational scheme, ρ(*x*) should be self-consistently calculated for a fixed displacement *x* of the oscillator through the following integral


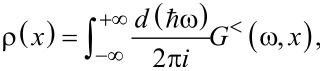


with the lesser Green’s function *G^<^*(ω,*x*) = (*i*/2)[*f_L_*(ω) + *f_R_*(ω)]*A*(ω,*x*). The above approximation is valid if the electron–oscillator interaction is not too large, such that 
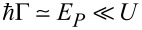
 and the peaks of the spectral function can be still resolved [82].

This section is intended to report a summary of the results presented in [82]. In this work, it has been shown that, in order to study the heat transport through a quantum dot junction at finite *U*, the above approximation for the electronic Green’s function is a reliable starting point for the adiabatic approach. The effect of the phonon leads has also been included in the adiabatic expansion and it has been shown that the oscillator dynamics is described by a Langevin equation with the same structure of that derived in section 2. Before discussing the numerical results, it is useful to analyze the properties of the electron–oscillator damping rate θ′(*x*) = θ(*x*)/*m*, with θ(*x*) defined in Equation 18 (explicitly calculated in [82]) and appearing explicitly in the Langevin equation of the oscillator. The amplitude of the peaks of θ′(*x*), although increasing as a function of the electron–oscillator coupling *E_P_*, is always smaller than the damping contribution (the realistic value γ = 0.15 is assumed in the second part of this section) coming from the coupling with the phononic leads. Therefore, regardless of its shape, which also depends on the dot occupation and obviously on the Coulomb repulsion *U*, θ(*x*) constitutes a small perturbation of the spatially constant damping rate induced by the coupling to the phononic leads.

**Figure 7:**
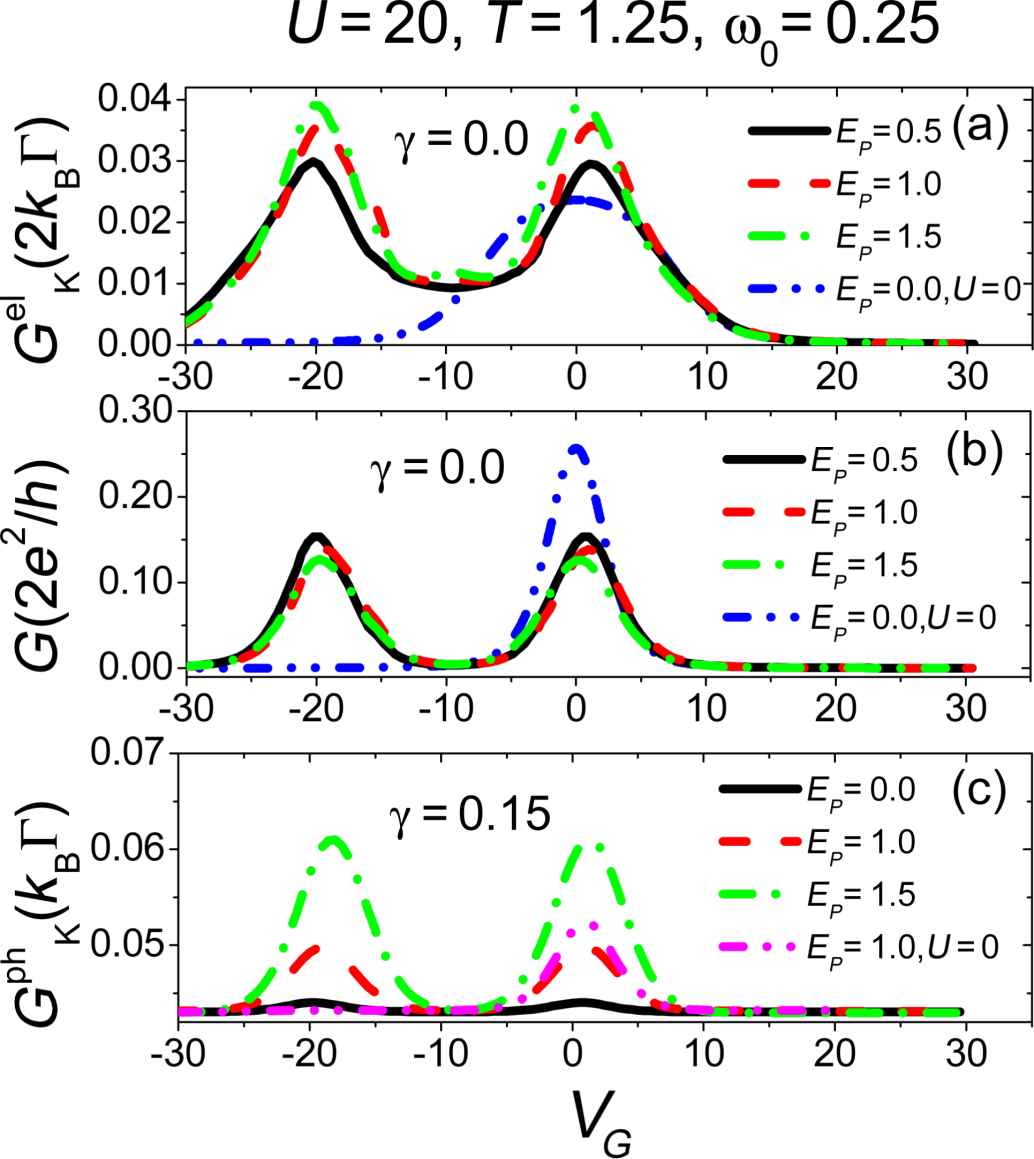
Thermal transport properties in the linear response regime, in the range of validity of the adiabatic approximation and including the electron–electron interaction. Panel (a): Electron thermal conductance 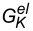 in units of 2*k*_B_Γ as a function of the gate voltage *V_G_* (in units of 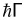) for different values of electron–vibration coupling *E_P_* (in units of 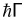). Panel (b): Same as in panel (a) for the electron conductance *G* in units of 2*e*^2^/*h*. Panel (c): Phonon thermal conductance 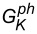 (in units of *k*_B_Γ) with the oscillator damping rate γ = 0.15Γ. Adapted and reproduced with permission from [82], copyright 2015 IOP Publishing.

**Figure 8:**
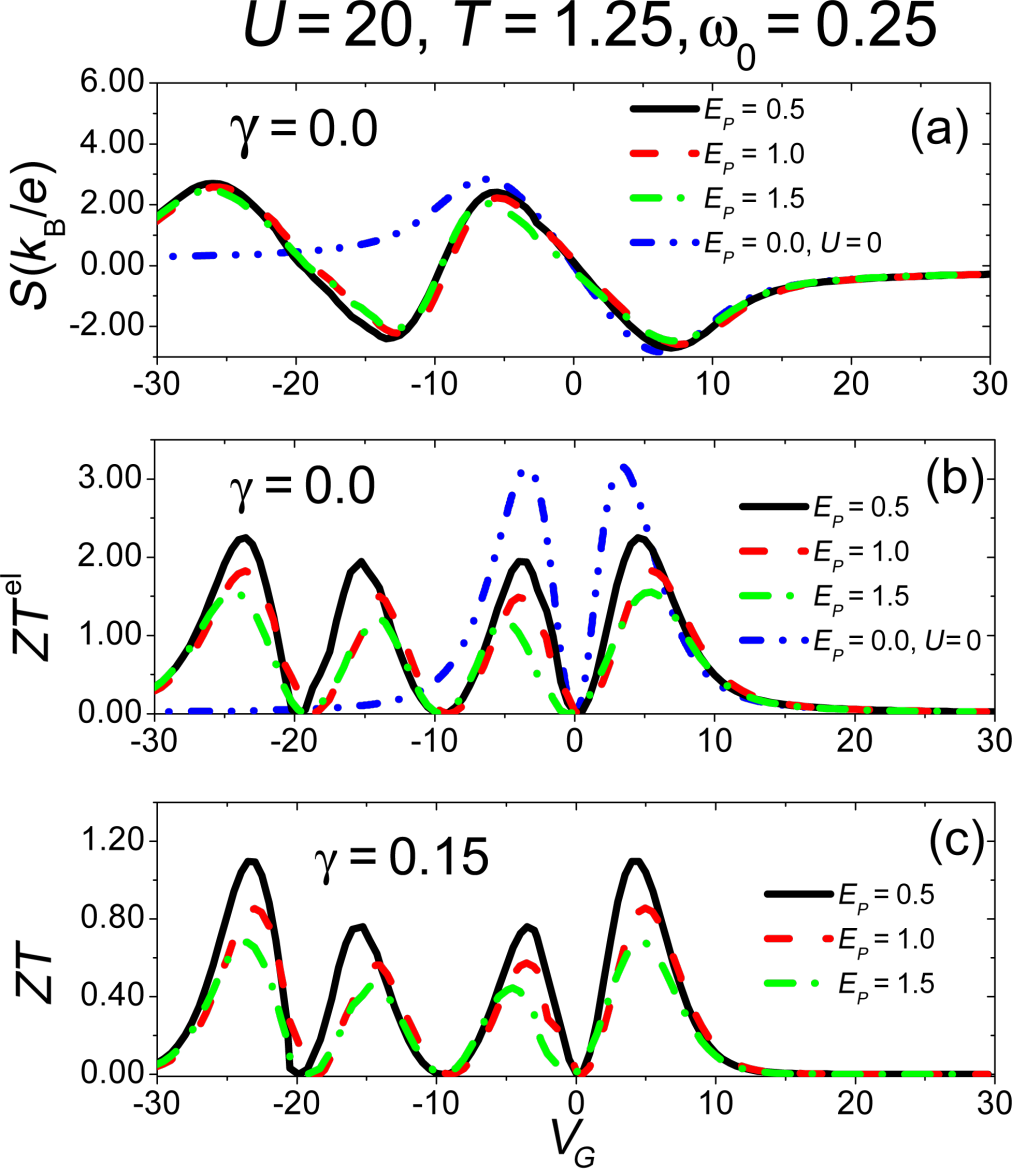
Thermal transport properties in the linear response regime, within the range of validity of the adiabatic approximation and including the electron–electron interaction. Panel (a): Seebeck coefficient *S* in units of *k*_B_/*e* for different values of electron–vibration coupling *E_P_* (in units of 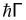). Panel (b): Dimensionless thermoelectric figure of merit *ZT^el^* for the electronic system only. Panel (c) shows the total thermoelectric figure of merit. Adapted and reproduced with permission from [82], copyright 2015 IOP Publishing.

In the following, unless otherwise stated, we consider a Coulomb repulsion equal to *U* = 20 (being the largest energy scale in the problem) and a value *T* = 1.25 mimicking a regime close to room-temperature conditions. In order to better distinguish thermal electronic effects from those related to the direct link between the vibrational molecular modes and the lead phonon bath, we first discuss briefly the thermoelectric electronic properties when γ = 0. In Figure 7a,b and in Figure 8, we study electronic properties of the quantum dot junction as a function of the electron–vibration coupling *E_P_* and the gate voltage *V_G_*. The dashed-dotted-dotted (blue) line indicate results obtained for *U* = 0 and no electron–oscillator coupling *E_P_* = 0. In Figure 7c the magenta dashed-dotted-dotted line indicates 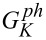 for *E_P_* = 1.0 and *U* = 0.0.

We start discussing the main characteristics of the electronic conductance *G* (Figure 7b) in linear response. Notice that, the height of the peak at *V_G_* 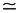 0 of the free case is much higher than the peak at non-zero electron–oscillator coupling. This effect is expected since the electron–electron interaction usually suppresses the electronic conduction. The height of the second peak at *V_G_* 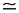 −20 has the same height of the first peak and can be estimated to be of the order of 10^−1^*e*^2^/*h* (*e*^2^/*h* is about 3.87 × 10^−5^ S). Away from the main peaks, *G* has values close to the experimental data estimation for a C_60_ junction [92]. The double peak shape of the *G*-vs-*V_G_* curve follows closely the behaviour of the *A*(ω,*x*). Finally, observe that the main effects of the electron–oscillator interaction are a reduction of the amplitude of the peaks, an increase of their broadening, and a shift their position towards negative energy.

We now investigate the properties of the Seebeck coefficient *S* of the junction in Figure 8a. Notice that *S* has a peculiar oscillatory behaviour as a function of *V_G_*, with positive peaks and negative dips. The height of the peaks (same as that of the dips in absolute value) is about 2*k*_B_/*e* (*k*_B_/*e* is about 86 μeV/K). Notice that the Seebeck coefficient *S* is negligible around the position where the electronic conductance exhibits the main peaks, that is at *V_G_* 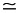 0 and *V_G_* 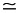 −20. This property is a result of the strong electron–electron interaction *U* [94]. Interestingly, for large positive values of *V_G_*, *S* is small and negative (n-type behaviour). In particular, for *V_G_* = 20, *S* can be estimated to be −0.45*k*_B_/*e* 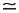 −38.5 μV/K, close in magnitude of the experimental data provided for a C_60_ junction in [92].

Another important property to mention in the comparison of *S* and *G* is that, away from the points *V_G_* 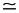 −20 and *V_G_* 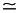 0, while *G* reduces its amplitude, *S* increases its amplitude in absolute value. Moreover, even in this case, the main effect of the electron–oscillator interaction is to reduce the amplitude of the response function, for all the values of *V_G_* investigated. The shift of the peaks of *G* and of the zeroes of *S* is governed by *E_P_* (*n* = 0.5 for *V_G_* = *E_P_*). We point out that, at fixed gate voltage, while the conductance *G* (Figure 7b) shows a variation of less than 10%, the Seebeck coefficient shows a larger sensitivity (between 10% and 20%) to the change of the coupling *E_P_* (see Figure 8a). This occurs for energies close to the minimum and the maximum. For larger values of *V_G_*, there is an inversion in the behaviour of *S* with increasing the coupling *E_P_*.

In Figure 7a, we also study the electronic thermal conductance 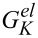 as a function of *V_G_*. The principal characteristic to mention is that, by increasing the electron–oscillator coupling *E_P_*, the height of the two peaks (again, similarly to the conductance *G*, notice the two-peak structure [94] of 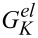) at *V_G_* 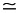 0 and *V_G_* 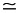 −20 increases. This is due to the opening of extra channels of conduction due the larger broadening of the spectral peaks. Indeed, for *E_P_* = 1, 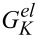 takes values of 0.05*k_B_*Γ 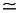 20 pW/K, which are close to those estimated experimentally in hydrocarbon molecules (50 pW/K) [95]. Notice that the height of the peaks (0.01*k*_B_Γ where *k*_B_Γ is about 4.198 × 10^−10^ W/K for 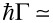 20 meV) are smaller than the thermal conductance quantum *g*_0_(*T*) = 
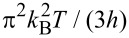
 at room temperature *T* = 1.25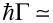 300 K (*g*_0_(*T*) 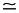 9.456 × 10^−13^ (W/K^2^)*T*) [96].

It is useful to observe the similarities between the 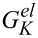 shown in Figure 7a and the phonon thermal conductance 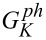 reported in Figure 7c. Indeed, they share a double peak shape and the property of increasing their amplitude with *E_P_*. Notice that the phonon thermal conductance 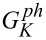 in Figure 7 has been obtained using γ = 0.15Γ and assumes in this case an amplitude comparable with 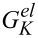. The amplitude of 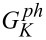 increases as a function of γ so a larger value of this quantity would imply a larger contribution to the total thermal conductance *G_K_*. Overall, at fixed gate voltage away from the peaks at *V_G_* 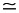 0 and *V_G_* 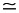 −20, 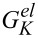 has an amplitude larger than 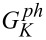.

The results described above concerning the electronic conductance *G*, Seebeck coefficient *S*, and thermal conductance 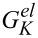 all combine in giving a pure electronic figure of merit *ZT^el^*. This latter quantity has been shown in Figure 8b. Notice the four-peak structure with amplitudes larger than 2. The dashed-dotted-dotted blue curve shows the results for *U* = 0, confirming that the effect of the electron–electron interaction is to reduce the amplitude and shift the peaks of the figure of merit, even neglecting the effect of the phononic leads. Increasing the electron–oscillator coupling *E_P_*, the behaviour of *G*, *S* and 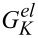 cooperates toward a further reduction of the height of the figure of merit peaks, which can assume a common average height smaller than 2. Observe finally that the position of the peaks in *ZT^el^* roughly coincides with the position of the peaks and dips of the Seebeck coefficient.

We can now study the behaviour of the thermal properties of the junction when also a coupling γ different from zero of the vibrational mode with the phononic leads is present. We begin discussing the phonon thermal conductance 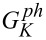 which has been already briefly described above in comparison with the electronic contribution 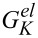. As observed early in this section, the 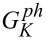 behaviour is strongly linked to the total damping rate of the oscillator. In the case where γ = 0.15Γ, the electron–oscillator contribution to the damping θ′(*x*) is smaller than the contribution of the phononic leads, so one expects that the behaviour of 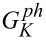 is mostly influenced by the electron–oscillator coupling *E_P_*. Indeed, this is what is observed in Figure 7c. If *E_P_* is not too large, the estimated height of peaks (as always at *V_G_* 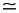 0 and *V_G_* 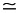 −20) of 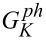 roughly coincides with an estimation of the same quantity in the absence of electron–electron and electron–oscillator interaction given in [86]. Furthermore, Figure 7c shows that the increase of the amplitude of 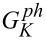 strongly depends on the gate voltage, while, at *E_P_* = 1, the height of the main peaks is 0.05*k*_B_Γ 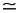 20 pW/K, which is close to the thermal conductance measured for molecules anchored to gold [95,97]. Summarizing, in the presence of a realistic coupling with the vibrational degrees of freedom of the leads, electron–electron interaction, and within the limits of validity of the adiabatic approximation, the phonon thermal conductance depends crucially on the rate of damping induced by the electron–oscillator coupling.

We finally explore the effect of the phonon thermal conductance on the total figure of merit *ZT*, which is plotted in Figure 8c as a function of the gate voltage with increasing values of the electron–oscillator coupling *E_P_*. Comparing with the results obtained for the pure electronic *ZT^el^*, one can see that the effect of 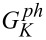 is to further reduce the amplitude of the figure of merit. The height of the peaks of *ZT* is slightly above unity for an intermediate coupling *E_P_* = 0.5, pointing to a reduction by a factor of 2 when compared with those observed in *ZT^el^*. If the electron–oscillator coupling is further increased, height of the peaks of the *ZT* can reduce to values below unity. We can therefore conclude this section observing that the combined effect of a large electron–electron interaction strength, an interaction with an adiabatic oscillator, and the presence of vibrational degrees of freedom in the leads, has the final result of reducing the thermoelectric capabilities of a molecular dot device. Nevertheless, it has been shown that for a large set of the model parameters, the total figure of merit *ZT* is still significant, pointing to possible future thermoelectric applications of these devices.

### 6 Time-dependent perturbations

#### 6.1 Suspended CNT with external antenna effects: electronic transport

**Figure 9:**
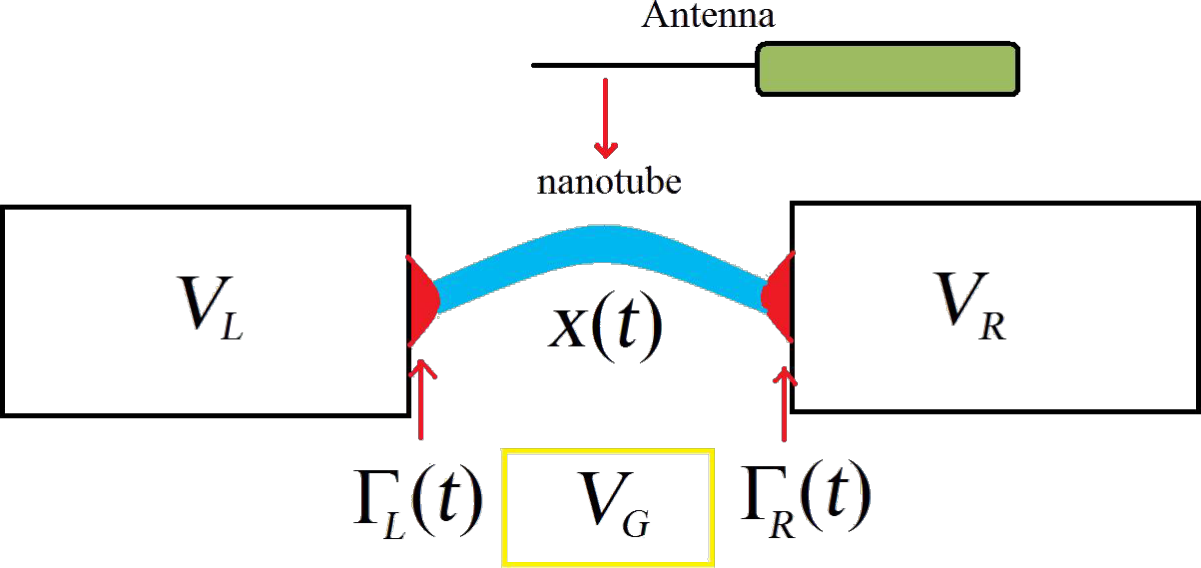
Sketch of the device investigated in section 6. A carbon nanotube is suspended between two metal leads to which a bias voltage *V_L_* = *−V_R_* = *V_bias_*/2 is applied. The motion of the nanotube is activated by an external antenna. The contact giving the gate potential *V_G_* is also shown. In the case of a two-parameter charge pumping, one has also a time-dependent modulation of the potential barriers between the leads and the nanotube, Γ*_L_*(*t*), and Γ*_R_*(*t*).

In this section we discuss the effect of the time-dependent perturbation induced by an external antenna on molecular devices. Our work has been motivated by recent transport experiments performed on suspended carbon nanotubes [11–12], where an external temporal periodic perturbation given by a nearby antenna actuates the flexural motion of the suspended nanotube. A sketch of the device studied in this section is reported in Figure 9. The main assumption is that the coupling between charge and vibrational degrees of freedom is affecting directly the mechanical displacement of the nanotube. In the general scheme outlined in section 1, the driving considered in this section can be described by (the semiclassical approximation is assumed)

[52]
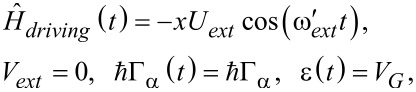


which means that in Equation 2, the dot 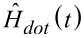 and the 
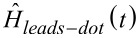
 parts are assumed to be independent of time and the driving is acting directly on the oscillator Hamiltonian in Equation 4, where the coupling with the vibrational degrees of freedom in the leads has also been neglected. Unless otherwise stated, in this and the next sections, we neglect the effect of a finite Coulomb repulsion *U*. We stress that in suspended carbon nanotubes devices, often a description in terms of a spinless electronic level is sufficient for capturing the main physics since the distance between adjacent electronic levels of the dot is assumed to be very large.

As thoroughly discussed by us [77,98], the regime of parameters relevant for the experiments is very accurately described by the adiabatic approximation for the vibrational degrees of freedom. Indeed, the vibrating nanotube is oscillating at a frequency in the megahertz range so that ω_0_/Γ 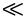 1, where Γ is the electronic tunneling rate. Also, a strong coupling between the electronic and vibrational degrees of freedom is realized in the experiments (*E_P_*/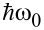 = 10), while for the other parameters one has 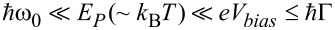. We observe here that the Langevin equation describing the dynamics of the CNT flexural motion acquires a forcing term

**Figure 10:**
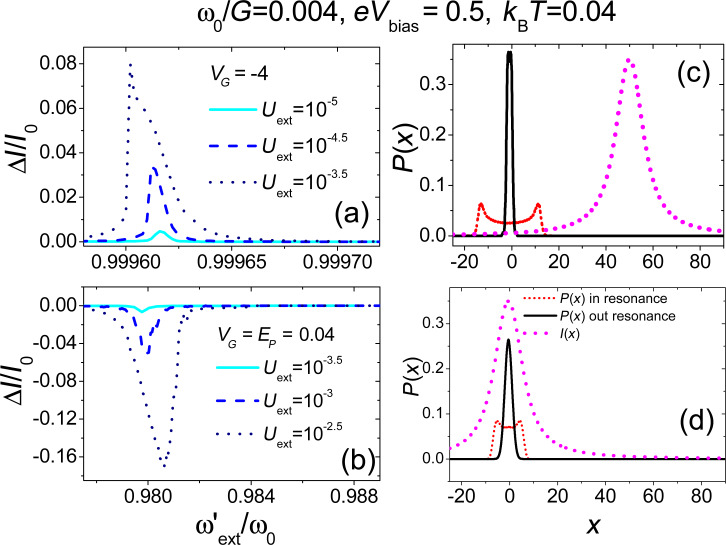
Electronic transport properties in the presence of an external periodic time-dependent perturbation. Panel (a) and panel (b): Normalized current change (Δ*I*/*I*_0_) as a function of the external frequency (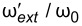) for different antenna amplitudes. Panels (c) and (d) show the distribution *P*(*x*) both out of mechanical resonance and at mechanical resonance for the larger value of antenna amplitude considered in panels (a) and (b). The short-dashed line (magenta) represents current as function of position *I*(*x*). Adapted from [77].

**Figure 11:**
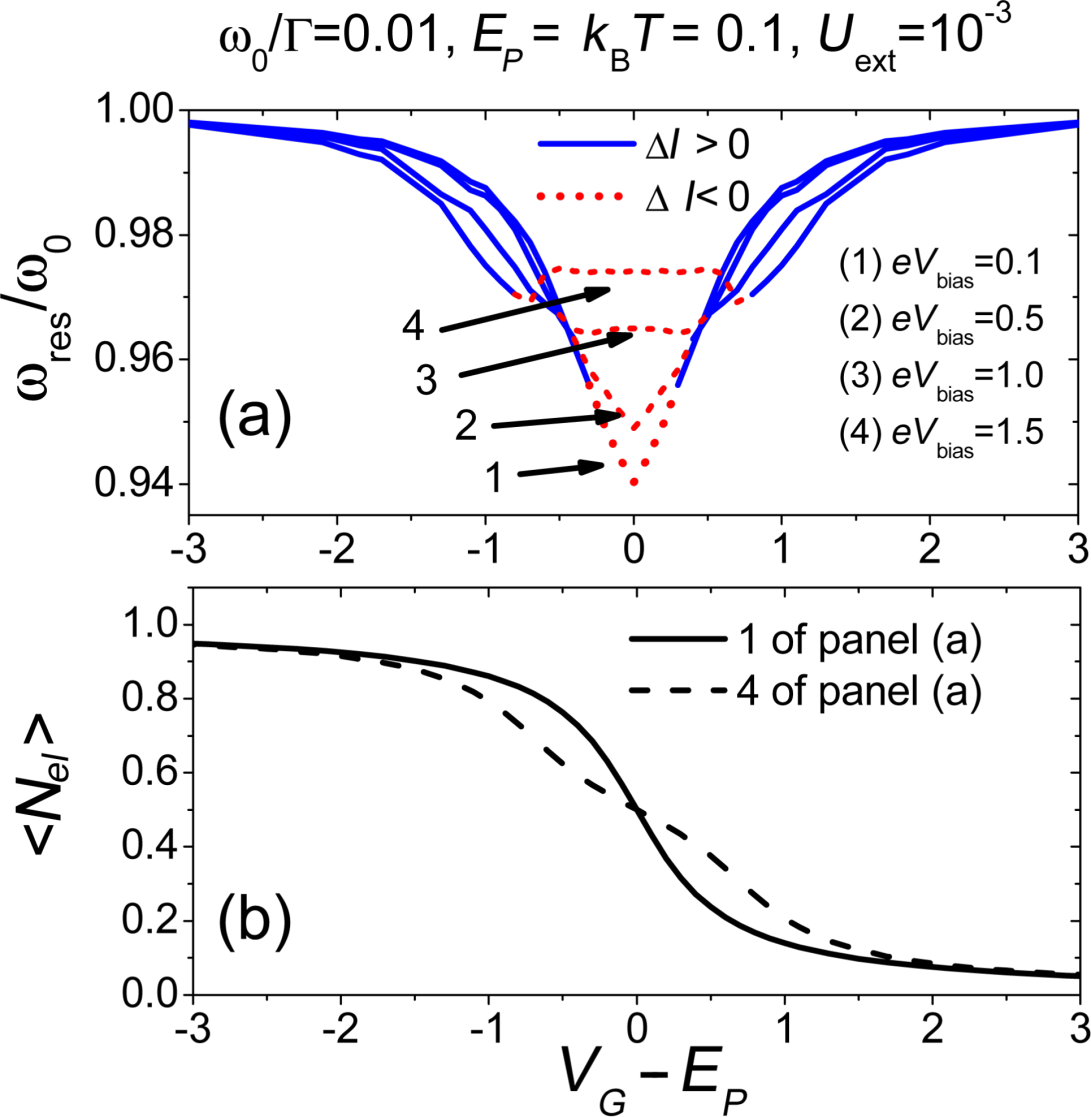
Interplay between the resonator frequency and the charge density. Panel (a): Resonator frequency at resonance against effective gate voltage (shifted of *E_P_*) for different bias voltages. Solid (blue) and short-dashed (red) portions of each curve indicate resonance frequency values with positive and negative current change Δ*I*, respectively. Panel (b): Electronic occupation at resonance frequency against effective gate voltage (shifted of *E_P_*). Adapted from [77].

[53]
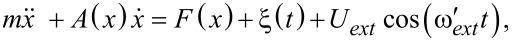


where *U_ext_* and 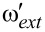 represent the amplitude and the frequency of the external antenna, respectively. In this section, the coupling with the phonon leads is neglected (γ = 0). In Figure 10 and Figure 11, we report a summary of the main results obtained by us in [77]. In this review, we intend to focus on the particular case when one tunes the frequency of the external antenna across the natural vibrational frequency of the oscillating nanotube.

In Figure 10a,b, we report the electronic current change (Δ*I*/*I*_0_, where Δ*I* = *I* − *I*_0_ and *I*_0_ is the current observed in the absence of external antenna) as a function of the antenna frequency 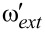 in two different regimes of the gate voltage applied on the nanotube. In panel (a), we show the case of low occupancy where a small current is flowing through the CNT (the external gate voltage with 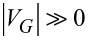 tunes the electronic level of the CNT away from the bias window), while in panel (b) a high current regime with *V_G_* = *E_P_* is shown. As already discussed by us in [77], a characteristic peak (panel (a)) and dip (panel (b)) structure is observed in qualitative agreement with the experimental results, including the particular shape of the curves reported when one increases the amplitude of the antenna field *U_ext_*.

A characteristic triangular shape is obtained in complete agreement with experimental results [11] accompanied by a shift in the position of the resonance frequency with respect to small amplitude regime. This nonlinear behaviour has been understood by analyzing the properties of the force *F*(*x*) (Equation 10) in the equation of motion, which has nonlinear terms stemming from the electron–vibration interaction. Softening, hardening and shape of the curves are usually related to the sign of the cubic nonlinear term in the force [99]. When the external gate voltage is tuned in such a way that the current flow through the device is blocked (
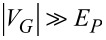
) the sign of this term is positive, giving a net softening effect. When the external gate voltage tunes the electronic level of the quantum dot within the conduction window (*V_G_* = *E_P_*) and for bias values sufficiently small, the sign of the cubic nonlinear term is negative providing an hardening.

In the experiments described in [11–12] it is has been shown that, under conditions of mechanical resonance, one can control the oscillation frequency of the nanotube by tuning the gate voltage. Motivated by this finding, in Figure 11a we review the results obtained by us in [77] concerning the natural frequency of the nanotube as a function of the gate voltage applied to the junction. As in the actual experiment, the position of the frequencies are detected by calculating the electronic current change (analysed in Figure 11 in the regime of small antenna amplitudes). The solid part of the curves (red) indicates that a positive current change peak was found (as in panel (a)), while the dashed part (blue) indicates that a negative current change was found (as in panel (b)).

If one observes curve (1) of Figure 11a, a characteristic V-shaped curve is found in almost quantitative agreement with experimental results in [11]. The observed renormalization of the resonance frequency can be related to the variations of the electronic occupation as function of the gate voltage (see solid line in Figure 11b). Increasing the bias voltage to values closer to 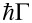 or larger (line (3) and (4) of Figure 11a), one obtains a non-trivial renormalization of the resonance frequency as function of the gate. We note that for *eV_bias_* = 1.5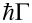 (line (4) of Figure 11a), a fine structure represented by two very small dips appears. This feature has been experimentally observed recently in [100].

#### 6.2 Single-parameter charge pumping

In this review, as stated before, we will discuss some of the effects that arise when an external temporal periodic driving gets resonant with the internal frequency of the quantum nanosystem. In this subsection, we consider a nano-system very similar to that considered previously but with a different coupling between the electric field produced by the external antenna and the quantum dot degrees of freedom. In particular, we assume that the coupling is affecting directly the electronic gate potential (*U_ext_* = 0 and *V_ext_* ≠ 0 in Equation 4).

The driving considered in this section can be described by

[54]
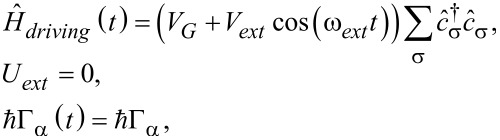


which means that in Equation 2, the 
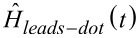
 part as well as the oscillator Hamiltonian in Equation 4 are assumed to be independent of time, while the coupling with the vibrational degrees of freedom in the leads has also been neglected even if in this section the electronic temperature of the leads is assumed to be non-zero.

As thoroughly discussed by us in [79], when the external antenna frequency is close to mechanical resonance with the natural frequency of the dot, one can observe a charge current flowing through the system realizing a single-parameter quantum pumping. We point out that the conventional quantum pumping can only be realized with two out of phase driving parameters [47,101–104], for example left and right lead voltages or one lead and the gate voltages.

**Figure 12:**
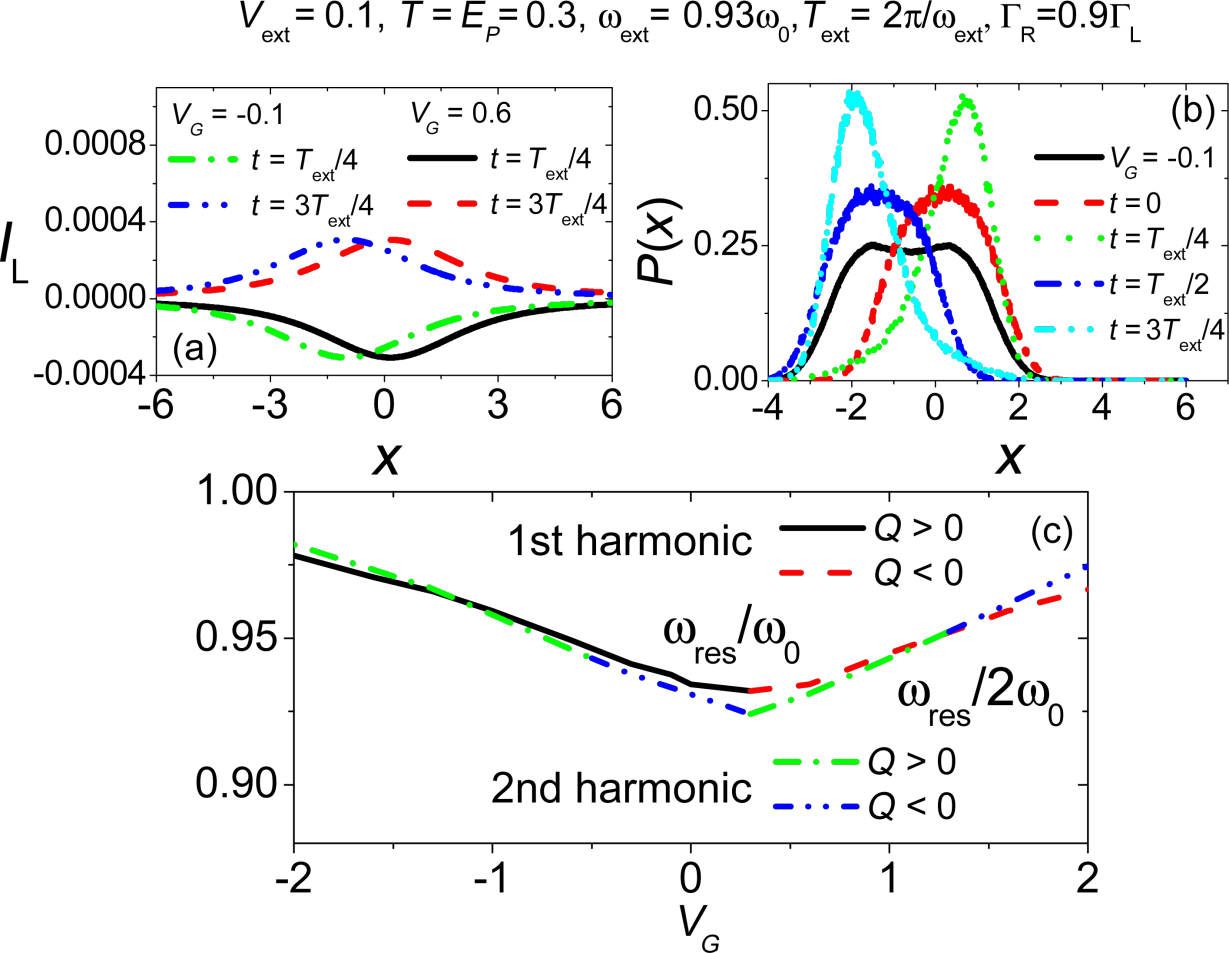
Single-parameter charge-pumping mechanism, and oscillator frequency as a function of the gate voltage. Panel (a): Left current *I_L_* as a function of oscillator position *x* for different times *t* and static gate voltages *V_G_*. Panel (b): Reduced position probability distribution *P* as a function of *x* for different times *t* at fixed static voltage *V_G_*. The time averaged distribution is in black line. Panel (c): Softening of the resonance frequency corresponding to the first and to the second harmonic as a function of the static gate voltage *V_G_*. Adapted and reproduced with permission from [79], copyright 2013 EPLA.

We hereby review [79] the main mechanism that allows for the single-parameter charge pumping in a system that can be realized by a suspended carbon nanotube quantum dot. In Figure 12a, we report the electronic current *I_L_*(*x*) flowing through the system as a function of the CNT vibrational displacement. In particular, we show *I_L_*(*x*) at a quarter and at three quarters of the period *T_ext_* for different values of static gate *V_G_*. As one can see, close to mechanical resonance, in the first half-period it shows a different behaviour from that in the second half-period (in the case where ω*_ext_* = 0.93ω_0_). This involves that the average over a period is different from zero, allowing to pump charge into the nanotube.

At fixed *V_G_*, *I_L_*(*x*,*t*) acquires a minimum at a quarter of a period, while a maximum at three quarters of a period. Moreover, the static gate induces a shift of the curves toward positive values for negative *V_G_*, but negative values for positive *V_G_*. The shifts of *I_L_* are compared with the behaviour of *P*(*x*,*t*) at the resonance. As shown in Figure 12b, in addition to the new center of the distribution due to the coupling *E_P_*, the distribution averaged over a period (black line) is bimodal due to the resonance phenomenon. In the supplementary material of [79], we point out that the bimodal character is present only close to the resonance. For the reasons explained above, the tail of the distribution probability *P*(*x*,*t*) is always able to intercept a spatial region where *I_L_* is not zero giving an average non-zero charge pumped through the nanotube.

We point out that the pumping mechanism is due to a relevant dynamical adjustment of the oscillator to the single external drive and cannot be understood in terms of a phase shift between the external drives, such as in the two-pumping parameter mechanism [47], or between the ac gate voltage and the parametrically excited mechanical oscillations [105]. Moreover, we have considered a configuration where the inversion symmetry has been broken: the coupling Γ*_L_* to the left lead is slightly different from the coupling Γ*_R_* (Γ*_R_* = 0.9Γ*_L_* = 0.9Γ). A small asymmetry is sufficient to induce a single-parameter pumping even if the system is in the adiabatic regime.

In analogy to what was considered under out-of-equilibrium conditions in the previous subsection, we here review our theoretical proposal [79] of controlling the oscillation frequency of the nanotube by tuning the gate voltage under conditions of mechanical resonance even at zero bias where pumping is realized. In Figure 12c, we show the natural frequencies of the nanotube as a function of the gate voltage applied to the junction. We point out that the particular coupling between the electronic and vibrational degrees of freedom allows us to excite also higher harmonics of mechanical vibration of the nanotube.

**Figure 13:**
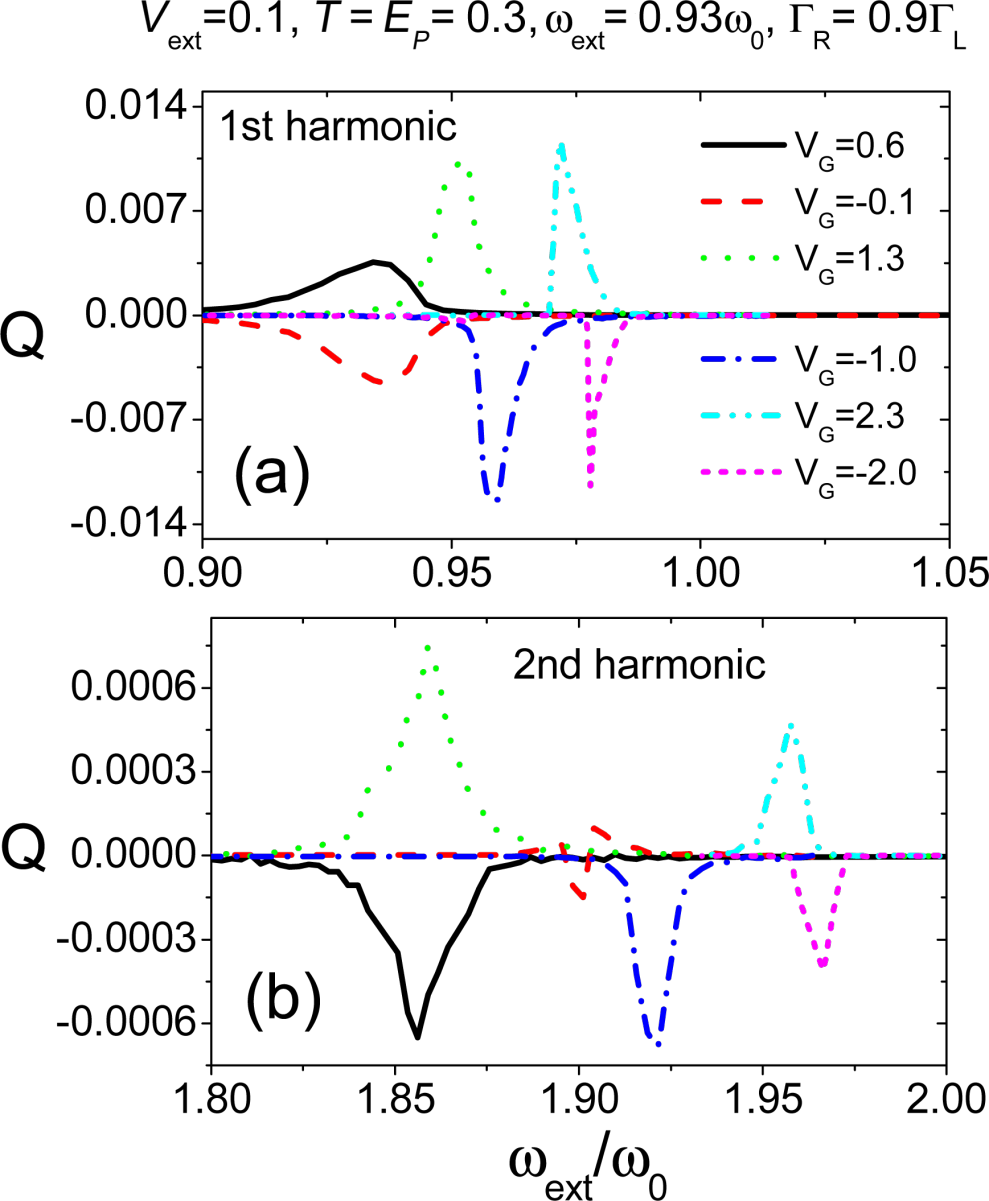
Single-parameter charge pumped at zero bias. Panel (a): Charge *Q* (in units of *e*) as a function of the external frequency in the interval close to 1 × ω_0_ with varying the static gate *V_G_*. Panel (b): Charge *Q* (in units of *e*) as a function of the external frequency in the interval close to 2 × ω_0_ with varying the static gate *V_G_*. Adapted and reproduced with permission from [79], copyright 2013 EPLA.

As in the experiments described in [11–12], the position of the natural CNT frequencies are detected by calculating the change pumped as a function of the antenna frequency (as in Figure 13) for different gate voltages. As shown in Figure 12c (*V_ext_* = 0.1, slightly nonlinear regime), the first harmonic resonance has a characteristic V-shape similar to that seen in the previous section for a different antenna–vibration coupling. We point out that the softening is symmetrical with respect to *V_G_* − *E_P_* even if the pumped charges have opposite signs (see below). We found that the characteristics (softening, hardening) of the second harmonic are similar to those of the first harmonic. Once the frequency of the first harmonic has been individuated, in order to explore the behaviour of the system at higher harmonics it is sufficient to tune the external antenna frequency close to integer multiples of the proper frequency *n* × ω_0_. We expect that higher harmonics, which could be experimentally excited by a larger antenna power, exhibit a similar behaviour.

We notice that in Figure 13a,b the sign of the pumped charge depends on that of *V_G_*. In fact, this is due to the different behaviour of the currents for positive and negative *V_G_*. As a result, there is a specular symmetry with respect to *V_G_* − *E_P_* (*E_P_* = 0.3 in Figure 12a,b). Moreover, with increasing *V_G_*, the shape of the charge–frequency curves tends to be more triangular as a function of the frequency, meaning that the response becomes progressively nonlinear with features of the Duffing oscillator [77,99]. In [79], we have studied the current–frequency response of the device by increasing the antenna power well above the linear regime. We observed that the pumped current at the first harmonic increases to a maximum, where the second harmonic response becomes appreciable. By increasing the antenna power further, the response at the first harmonic reduces while the second harmonic increases up to a maximum value as the first harmonic. The response is successively transferred to higher harmonics, and eventually the total pumped charge changes sign. We finally discuss the response for frequencies close to second harmonic. As shown in Figure 13b, in the weakly nonlinear regime (*V_ext_* = 0.1), some charge (less than 10% of that corresponding to the first harmonic) is pumped close to those frequencies. Moreover, the frequency response shows a very complex behaviour with several maxima and minima that should be experimentally observed in future experiments.

#### 6.3 Noise-assisted pumping

In this section, we investigate another nano-system where charge pumping effects can be reinforced and amplified against temperature and noise when one excites the system close to the mechanical resonance with an external driving. In particular, we here review some of recent results obtained by us in [84]. We consider a conventional quantum pumping scheme [101] where the tunneling amplitudes between the dot and the leads (*u*_α_(*t*) represents the strength of the pumping) are oscillating periodically in time due to external fields. We consider that no antenna effect and no bias voltage is present with γ = 0 (no coupling with phonon leads).

In the general scheme outlined in section 2, the driving considered in this section can be summarized in the following equation

[55]
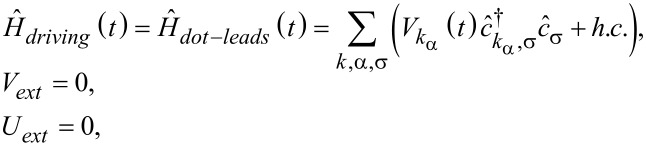


which means that in Equation 2, the dot part 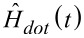 is assumed to be independent of time (ε(*t*) = *V_G_*) and the driving is acting directly on the coupling between the dot and the leads. In particular, 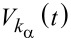 = 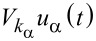 giving 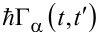 = 
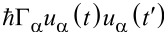
, where *u*_α_(*t*) = 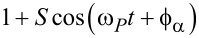, with *S* being amplitude of the pumping driving with the frequency ω*_P_* and phase 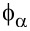, with phase shift 
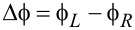
. Finally, the oscillator Hamiltonian is assumed to be independent of time.

In order to apply the adiabatic approximation, we have assumed that the external time-dependent perturbations are slowly varying in time together with the mechanical mode vibrational motion: 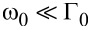 and 
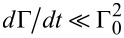
, where we have assumed that Γ*_L_* = Γ*_R_* = Γ_0_.

In [84], we have shown that the adiabatic approach leads to a Langevin dynamics for the vibrational mode where the external fields give rise to a forcing term as in the case of an antenna. Indeed, the deterministic part of the force appearing in the Langevin equation contains also a dissipative term proportional to the velocity

[56]
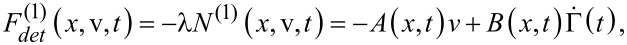


with the coefficients *A*(*x*,*t*) (positive definite) and *B*(*x*,*t*) taken from [84]. Remarkably, we have also verified that the noise strength associated with this force fulfills the fluctuation–dissipation theorem at each oscillator position and time *D*(*x*,*t*) = 2*k*_B_*TA*(*x*,*t*), where the position-dependent noise *D*(*x*,*t*) has been calculated in general in Equation 28.

From the solution of the Langevin equation, one can calculate the oscillator distribution function *P*(*x*,*v*,*t*) and compute all the observable quantities as in the previous section. As stated above, in the regime of adiabatic pumping, one has 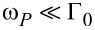, and 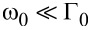, so that the dimensionless ratio *r_P_* = ω*_P_*/ω_0_ is of the order of unity. The regime of weak pumping is defined by the condition 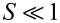, where *S* is proportional to the amplitude of the pumping terms. Throughout the section, we will assume ω_0_ = 0.1Γ_0_.

**Figure 14:**
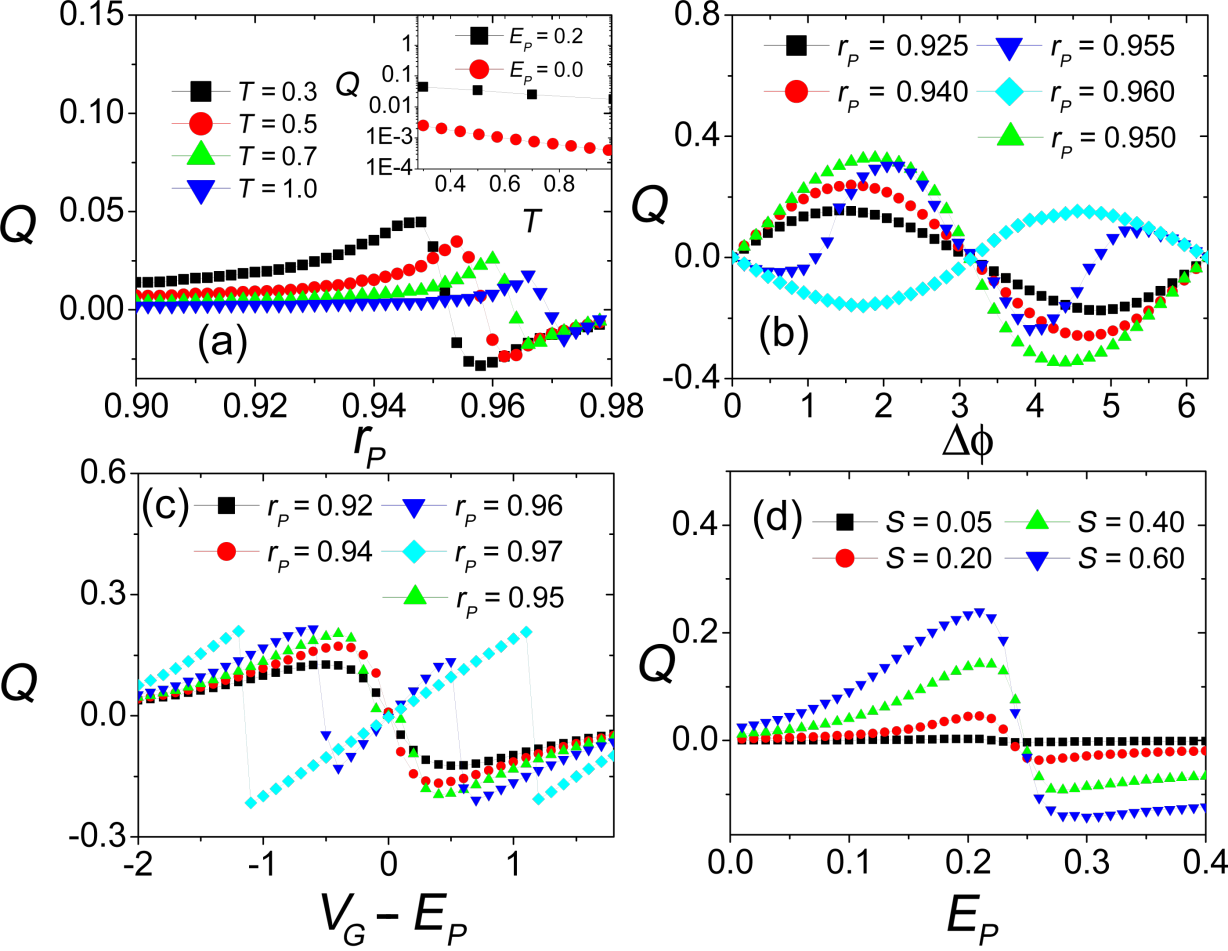
Electronic charge pumped in a two-parameter pumping device in the presence of an adiabatic vibrational degree of freedom. Panel (a): The pumped charge *Q* as a function of the external frequency for different temperatures. Inset: The value of the charge at the maximum as a function of the temperature is compared with the pumped charge for *E_P_* = 0. In this panel *T* = 0.3, *V_G_* = −0.1, *S* = 0.20, 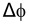 = π/4. Panel (b): The pumped charge *Q* for *S* = 0.5 as a function of the phase difference 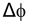 for different values of *r_P_*. In this panel *T* = 0.3, *V_G_* = −0.1, and *E_P_* = 0.2. Panel (c): The pumped charge *Q* as a function of *V_G_* − *E_P_* for different values of *r_P_*. In this plot *T* = 0.3, *V_G_* = −0.1, *E_P_* = 0.2, *S* = 0.50, and 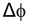 = π/4. Panel (d): The pumped charge *Q* as a function of the electron–oscillator coupling *E_P_* for different values of the pumping strength *S*. In this plot *T* = 0.3, *V_G_* = −0.1, *r_P_* = 0.945, and 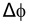 = π/4. Notice that the pumping strength *S* = 0.6 corresponds to the driving ratio 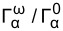 ≈ 1.765, which is very close to the maximal value of 2. Adapted and reproduced with permission from [84], copyright 2014 IOP Publishing.

In Figure 14a, we plot the pumped charge 
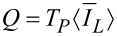
 as a function of the pumping frequency ω*_P_*, hence as a function of *r_P_* for different temperatures. We point out that 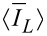 is the average current over one period of the driving 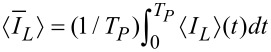, where 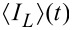 has been obtained averaging over *P*(*x*,*v*,*t*).

As one can see, the curves show a characteristic peak–dip structure around the renormalized frequency *r_eff_* = ω*_eff_*/ω_0_, where the charge pumped through the system is zero. This compensation effect (also observed in [106]) is due to a non-trivial dynamical adjustment of the vibrational mode distribution probability against the temporal variation of the current density in the phase space [84]. Note that, due to the electron–oscillator interaction, a strong softening of the bare frequency is expected as a function of the system parameters [77,107]. As shown in the inset of panel (a), this fact affects the behaviour of the pumped charge as a function of the temperature. Even if decreasing with temperature, the pumped charge under resonance conditions is always larger than the same quantity in the absence of interaction with a vibrational degree of freedom.

Another key element to explain the mechanism of cooperation between the vibrational and electronic degrees of freedom in the pumped charge is nonlinearity. In order to amplify the nonlinearity effects, in Figure 14b we show the pumped charge *Q* at pumping strength *S* = 0.5 as a function of the phase difference 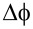. Different values of the external frequency ω*_P_* are shown. Away from the resonance regime (*r_P_* = 0.950), the response has a perfect sinusoidal shape, meaning that only the first harmonic is contributing. In resonance conditions, the response is distorted by the contribution of many harmonics.

In Figure 14c we show the pumped charge *Q* as a function of *V_G_* for different values of *r_P_*. We note an interesting threshold behaviour as a function of the frequency of the pump. Indeed, the curves correspondent to *r_P_* = 0.92, *r_P_* = 0.94 and *r_P_* = 0.95, which satisfy the condition *r_P_* ≤*r_eff_*, where *r_eff_* = 0.95 for 
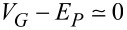
, show a change of sign just at 
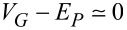
. Differently, for *r_P_* = 0.96 and *r_P_* = 0.97, *Q* suddenly changes sign at finite values of *V_G_* − *E_P_*. For larger values of *r_P_* (not shown in Figure 14), the pumped charge flattens, then, for *r_P_* close to unity, it tends to small negative values. Even in the presence of electron–oscillator interaction, when the gate voltage is tuned very far from the conduction window (
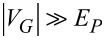
) the softening of the oscillator frequency tends to zero in agreement with Equation 9 of [106]. Finally, in Figure 14d we study the pumped charge *Q* as a function of the electron–oscillator coupling *E_P_* for different values of the pumping strength *S*. We notice that, around the intermediate coupling *E_P_* = 0.2, the pumping charge has, in absolute value, the maximum increase as function of the pumping strength. Also, for *S >* 0.05, an interesting change of sign in the pumped charge at 
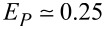
 occurs.

## Conclusion

In conclusion, we have generalized the adiabatic approach for nanoscopic systems in the presence of slow vibrational degrees of freedom to the case where time-dependent perturbations are acting on the system. Focus has been on the prototype model system consisting of a single electron level with a slow single vibrational mode in the parameter regimes appropriate to different soft nanosystems, such as molecular junctions and NEMS.

In this work, we have identified the range of parameters where the adiabatic approach is reliable in the absence of time-dependent perturbations. We have constructed the phase diagram (Figure 4) of the model in the presence of an applied finite bias voltage at zero temperature. The average kinetic energy of the vibrational mode is shown to play a crucial role in the establishing the validity of the method (Figure 3b). Its meaning is related to the effective excitation energy of the vibrational modes dynamically induced by a bias voltage or a temperature gradient. When this quantity is larger than the static vibrational energy of the modes, the adiabatic approach can be meaningfully applied to study the charge or heat transport.

At zero bias voltage and finite temperature, a comparison with a calculation which is exact in the low charge density limit on the dot has shown that the semiclassical adiabatic approach describes accurately the device down to quite low temperatures (Figure 2).

We have studied the current–voltage characteristic at zero temperature (Figure 5) and have observed a complete cancellation of hysteresis or finite discontinuity jumps typical of the static infinite mass approximation.

For sufficiently large electron–oscillator interaction strength, contrary to the expectations, we find a region of the parameter space where the kinetic energy decreases as a function of the bias voltage. Correspondingly, a finite electronic current flow is observed in the device, contrary to the static limit where it was completely blocked.

We have studied the thermoelectric properties within the linear response regime at room temperature (Figure 6). In particular, we have analyzed the role played by the phonon thermal contribution 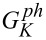 on the thermoelectric figure of merit *ZT* in the presence of electron–vibration coupling. We have found that 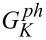 is of the same order of the electronic thermal conductance 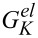 and it gets larger with increasing the electron–vibration coupling. Moreover, deviations from the Wiedemann–Franz law are progressively reduced with increasing the electron–vibration coupling. Therefore, the figure of merit *ZT* depends appreciably on the behaviour of 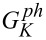 and electron–vibration coupling. Indeed, for realistic parameters of the model, *ZT* can be substantially reduced, but it can still have peaks of the order of unity with enhancements due to temperature increase.

We have then included the effect of a strong local repulsive electron–electron interaction, addressing the thermal transport in the Coulomb blockade regime (Figure 7 and Figure 8). Within the intermediate electron–vibration coupling regime, the phonon thermal conductance 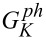 has a behaviour similar to the electron thermal conductance 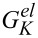. With increasing the electron–vibration coupling, they both get larger as in the absence of electron–electron interaction, while the charge conductance *G* and the thermopower *S* get smaller. The main result is that the figure of merit *ZT* depends considerably on the behaviour of 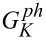 and intramolecular interactions. Indeed, for realistic parameters of the model, *ZT* can be substantially reduced, but its peak values can be still of the order of unity indicating that our results can be very interesting for applications.

In the presence of time-dependent perturbations, we have shown that the vibrational modes are driven in dynamical states that can be very well described in terms of our adiabatic approach. In particular, we have studied a single-level quantum dot realized by a suspended carbon nanotube including, in a non-perturbative way, the effect of the antenna actuating the nanotube motion (a schematic illustration of the device is given in Figure 9). For the scope of this review, we have reproposed [77] the main features of the device when the antenna drives the system close to the mechanical resonance with the natural vibrational frequency (Figure 10 and Figure 11). The current–frequency curves have been studied, showing a very good agreement with the experimental results. Here the nonlinear effects are understood without adding extra nonlinear terms to the effective force exerted on the resonator [12,98,108], but they are shown to be naturally included in our adiabatic scheme.

In the presence of the same antenna, for frequencies close to the mechanical resonance, we have shown that it is possible to realize single-parameter adiabatic charge pumping (Figure 12 and Figure 13). The mechanism [79] is different from that active in the two-parameter pumping since it requires a dynamic adjustment of the mechanical motion of the nanotube to the external drive. Moreover, the excitation of the second harmonic is feasible showing a similarity of the softening with the first harmonic.

Finally, we have studied the two-parameter quantum pumping through a molecular level coupled to a slow vibrational mode (Figure 14). Again, we have studied the device close to resonance conditions, showing that in this regime the presence of dissipation and noise does not destroy the pumping mechanism and, even, amplifies it. One of the main results has been the observation of reinforcement of the charge pumping as a function of the temperature close to resonant condition with respect to the situation where no vibrational motion of the dot is allowed. Furthermore, we have observed finite jumps in the charge *Q* vs gate voltage curves at finite values of *V_G_*, and an amplification of charge pumping by increasing the strength of the driving. These effects could be observable in future experiments.

In the future, our approach could be extended to the study of the thermal transport away from the linear regime in NEMS of molecular junctions in the presence of time-dependent perturbations [30–31]. New directions in the field include also the possibility to control directly the vibrational degrees of freedom in order to manipulate heat flow by use of time-varying thermal bath temperatures or various other external fields [109].

Summarizing, we have discussed common features of different soft nanosystems, such as molecular junctions and NEMS, under external drive. The effects induced by time-dependent perturbations are very marked when the external forcing is nearly resonant with the vibrational modes. Indeed, close to the mechanical resonance, the external temporal perturbations induce nonlinear regimes where the interplay between electronic and vibrational degrees of freedom plays a major role. We believe that our work could represent a guide for future studies of more realistic models of multi-level electronic systems coupled to many slow vibrational degrees of freedom, in the presence of time-dependent perturbations, such as pumping and external forcing antennas.
